# Panduratin A Inhibits TNF Alpha-Stimulated Endothelial Cell Activation Through Suppressing the NF-κB Pathway

**DOI:** 10.3390/biom15010034

**Published:** 2024-12-30

**Authors:** Kriangkrai Kiatsoonthon, Nitchakarn Phimthong, Saranyapin Potikanond, Nitwara Wikan, Wutigri Nimlamool

**Affiliations:** 1 Department of Pharmacology, Faculty of Medicine, Chiang Mai University, Chiang Mai 50200, Thailand; kriangkrai_kiats@cmu.ac.th (K.K.); nitchakarn_ph@cmu.ac.th (N.P.); saranyapin.p@cmu.ac.th (S.P.); 2 PhD’s Degree Program in Pharmacology, Department of Pharmacology, Faculty of Medicine, Chiang Mai University, Chiang Mai 50200, Thailand

**Keywords:** endothelial activation, VCAM-1, ICAM-1, monocyte adhesion, atherosclerosis, TNF-alpha, NF-κB, panduratin A

## Abstract

Upon exposure to inflammatory stimuli including TNF-α, endothelial cells are activated leading to the adhesion of monocytes to their surface. These events are involved in the pathophysiology of atherosclerosis. Since TNF-α activates the NF-κB pathway, which contributes to atherosclerosis, targeting this signaling pathway may help prevent the risk of developing the disease. The current study elucidated the inhibitory effect of panduratin A (PA) on TNF-α-induced endothelial activation and monocyte adhesion. We discovered that PA reduced the level of pro-inflammatory cytokine IL-6 and chemokine MCP-1 in the media collected from endothelial cells stimulated with TNF-α. In addition, PA inhibited the expression of ICAM-1 and VCAM-1 on the surface of TNF-α-induced endothelial cells resulting in a decrease in the number of monocytes attached to endothelial cell surface. Mechanistically, PA prevented IκB degradation and specifically suppressed NF-κB phosphorylation and nuclear translocation in endothelial cells. However, PA had no inhibitory effect on the phosphorylation of AKT, ERK1/2, p38, and JNK. Taken together, PA blocked the production of cytokine and chemokine, adhesion molecules, and monocyte adhesion in response to TNF-α stimulation, in part, through NF-κB inhibition. Our study suggests that PA may possibly be effective in blocking the pathophysiology of atherosclerosis.

## 1. Introduction

Atherosclerosis is found to be a leading cause of mortality worldwide. The etiology of this disease is multifactorial since many risk factors including smoking, diabetes mellitus, hypertension, and dyslipidemia can contribute to the disease. The pathology is primarily driven by chronic vascular inflammation characterized by the adhesion and diapedesis of mononuclear cells through the endothelium. Lipid-laden macrophages within the subintimal space are critical to plaque formation, which may result in endothelial dysfunction [1]. Endothelial dysfunction and vascular inflammation lead to morphological and functional alterations in the endothelium, termed endothelial activation, and tumor necrosis factor-alpha (TNF-α) along with other proinflammatory cytokines released from damaged or immune cells, further induce this activation [2].

In endothelial cells, upon TNF-α stimulation, IκB is targeted to be degraded, releasing NF-κB to be phosphorylated and engage in inflammatory protein synthesis. TNF-α signaling activates NF-κB, which triggers the upregulation of intercellular adhesion molecule-1 (ICAM-1) and vascular cell adhesion molecule-1 (VCAM-1). TNF signaling can engage additional pathways, including the activation of phosphatidylinositol-3 kinase (PI3K) and protein kinase B (AKT) in certain cell types [2,3,4]. Following endothelial activation, monocyte chemoattractant protein-1 (MCP-1), a potent chemokine, is released, promoting the recruitment of immune cells from the bloodstream to the lesion site. This upregulation enhances inflammatory cell binding and extravasation into the subendothelial space, contributing to plaque formation [2,5]. Moreover, many cytokines including IL-1β and IL-6 are produced by cell types relevant to atherothrombosis, including immune and endothelial cells. Inhibition of these cytokines has been reported as a novel method for cardiovascular disease protection [6,7].

Currently, statins, HMG-CoA reductase inhibitors, are commonly used to prevent and treat atherosclerosis by lowering cholesterol synthesis and reducing serum LDL levels. However, statins also carry risks of serious side effects. For instance, the drug may cause renal toxicity, hepatotoxicity, new-onset type 2 diabetes, and statin-associated muscle symptoms (SAMs) [8,9]. Given these limitations, alternative strategies to reduce inflammation are being explored. Traditional agents such as non-steroidal anti-inflammatory drugs and immunomodulating agents, can have systemic side effects and pose cost limitations [10,11].

Phytotherapy has emerged as a potential alternative for managing these conditions. *Boesenbergia rotunda*, an herb used in Thai traditional medicine, exhibits various properties, including antimicrobial, antioxidant, antitumor, and anti-inflammatory effects. One of its active compounds, panduratin A (PA), has demonstrated strong inhibitory effects on inflammatory processes [12]. In particular, PA has been shown to suppress mRNA expression and the release of cytokines while blocking NF-κB activation in microglial cells [13]. It has also demonstrated anti-inflammatory effects in periodontal models activated with lipopolysaccharide (LPS) [14]. Moreover, the compound was reported to inhibit NF-κB translocation in TNF-treated A549 lung cancer cells in a dose-dependent manner [15].

However, there is no study investigating the modulatory effects of PA on activated endothelial cells. Therefore, we aimed to explore the inhibitory effects of PA on TNF-α-activated endothelial cells and define its responsible molecular mechanism of action.

## 2. Materials and Methods

### 2.1. Cell Culture

HMEC-1 (CRL-3243 ™) endothelial cells were bought from ATCC (Manassas, VA, USA) and maintained in MCDB131 medium (Thermo Fisher Scientific, Waltham, MA, USA) added with fetal bovine serum (10%), glutamine (10 mM), hydrocortisone (1 µg/mL), and epidermal growth factor (10 ng/mL) (all supplements obtained from Thermo Fisher Scientific). Human monocytes, THP-1 (TIB-202™) were also purchased from ATCC and maintained in complete medium, which was RPMI-1640 medium (Thermo Fisher Scientific) added with 0.05 mM 2-mercaptoethanol (Thermo Fisher Scientific) and 10% FBS. Remarkably, the 2-Mercaptoethanol was freshly added to the medium prior to seeding or performing fluid additions. HMEC-1 and THP-1 cells were cultured at a standard condition (37 °C and 5% CO_2_).

### 2.2. Cytotoxicity Analysis

HMEC-1 cells were treated with panduratin A (Sigma-Aldrich, St. Louis, MO, USA) at 0 to 10 μM (or with DMSO as a vehicle control) for 48 h. Then, conditioned media were discarded, and cells were incubated with MCDB131 media containing MTT reagent (0.5 mg/mL) (Sigma-Aldrich) for 1 h in an incubator (at 37 °C and 5% CO_2_). After that, the MTT reagent solution was discarded, and the attached cells were gently washed by PBS 3 times before adding DMSO (100%) to solubilize the formed formazan. The signal was read at 570 nm by a microplate analyzer (BioTek Instruments, Winooski, VT, USA).

### 2.3. Cytokine and Chemokine Assay

Endothelial cells were cultured in 24-well plates treated with PA (2, 3, and 4 μM) or DMSO for 3 h. Then cells were induced with TNF-α (at 10 ng/mL) (Thermo Fisher Scientific) and incubated for 24 h. The cell culture supernatants were collected by centrifugation at 10,000 rpm, 4 °C, for 5 min, and subject to enzyme-linked immunosorbent assay (ELISA) to measure the level of IL-6 and MCP-1 using an ELISA kit (MAXTM Deluxe Set) (BioLegend, San Diego, CA, USA). First, immunoplates were incubated with the first antibody at 4 °C overnight and washed 3 times with a PBST-based washing solution. Next, the plate was added to the blocking solution for 1 h before incubating with the conditioned media for 2 h at room temperature (RT). After washing, the plate was incubated with the second antibody for 1 h at RT followed by an avidin–HRP solution for 30 min at RT. Then TMB substrate was added for 20 min at RT before the addition of a stop solution. The color signal was measured (absorbance at 450 and 570 nm) by an ELISA reader (BioTek Instruments).

### 2.4. Immunofluorescence Study

The presence of specific proteins at particular cellular compartments in endothelial cells was visualized by the immunofluorescence technique. Cells were cultured and allowed to adhere on glass coverslips placed in individual wells of 24-well plates. For detecting VCAM-1, ICAM-1, α-tubulin, β-catenin, and vimentin, cells were left untreated, induced with TNF-α, or induced with TNF-α + 4 μM of PA for 24 h. For detecting phosphorylated NF-κB (pNF-κB) and IκB, cells were treated with PA (4 μM) 3 h prior to induction with 10 TNF-α for 30 min. After treatment, cells on each coverslip were incubated with formaldehyde solution (4%) for 15 min at RT to fix the cells. Next, the fixed cells were permeabilized with a permeabilizing solution (0.3% TritonX-100 in phosphate buffer saline (PBS)) for 5 min at RT with gentle agitating. Then, non-specific binding was blocked by adding bovine serum albumin solution (1%) for 1 h at RT. Cells were incubated with primary antibodies (Cell Signaling Technology, Danvers, MA, USA) at 4 °C overnight. Next, cells were incubated for 1 h at RT with a blocking solution containing a secondary antibody (an anti-rabbit IgG conjugated with Alexa488) (Thermo Fisher Scientific, Waltham, MA, USA) and DAPI (4′,6-diamidino-2-phenylindole) (Cell Signaling Technology) for staining the nuclei. In some experiments, cells were counterstained with DyLight-594-Phalloidin to detect filamentous actin (F-actin) (Cell Signaling Technology). A Leica DMi8 Thunder Imager 3D Assay (Leica Microsystems Ltd., Wetzlar, Germany) was used to visualize and record the micrographs. The image analysis was performed by LAS X image-processing software version 3.8.1 (Leica Microsystems Ltd.).

### 2.5. Monocyte Adhesion Assay

Endothelial cells were cultured in a 96-well plate for 24 h. The cells were left untreated or treated with TNF-α (with or without the presence of 4 μM of PA or DMSO) for 24 h. Viable THP-1 cells were pre-labeled with a cell-permeant nuclear stain, Hoechst 33342 (Cell Signaling Technology), at 1 μg/mL in RPMI complete medium for 30 min. After washing by gentle centrifugation (130× *g* for 7 min), THP-1 cells (at an equal cell concentration) were gently layered over the endothelial monolayers in each well and incubated without agitation for 30 min at 37 °C with 95% air, 5% CO_2_. Unbound THP-1 cells were discarded during washing 3 times with a complete medium. Fixation was performed by incubating with formaldehyde (4%) for 15 min at RT. Following 3 washes, adherent THP-1 cells on endothelial cells were visualized and counted by using a live-cell imager (BioTek Lionheart FX automated microscope with Gen5 imaging software version 3.12.08) (Agilent Technologies, Inc., Santa Clara, CA, USA).

### 2.6. Western Blot Analysis

Endothelial cells were cultured in a 24-well plate for 24 h. Treatment of cells with PA at different concentrations (0–4 μM) or DMSO in serum-free media for 3 h prior to stimulation with TNF-α for 30 min. Cells were lysed using Laemmli SDS-sample reagent and heated at 95 °C for 5 min. In some experiments, nuclear and cytoplasmic extracts were prepared by resuspended in buffer A (10 mM Hepes, pH 10, 1.5 mM MgCl_2_, 10 mM KCl, 0.5 mM dithiothreitol (DTT), 300 mM sucrose, 0.1% Nonidet P-40). Next, cells were centrifuged (15,000× *g*) for 10 min at 4 °C, and the cytoplasmic extracts were collected. The remaining nuclear pellet was washed with cold PBS, and centrifuged (15,000× *g*) for 5 min at 4 °C. Proteins were separated and transferred by SDS-PAGE and electroblotting, respectively. After blocking, membranes were incubated with primary antibodies for detecting IκB, NF-κB, pNF-κB, AKT, pAKT, ERK1/2, pERK1/2, p38, pp38, JNK, and pJNK (Cell Signaling Technology) at 4 °C overnight. Cells were incubated with appropriate secondary antibodies (an anti-mouse IgG conjugated with IRDye^®^ 800CW or an anti-rabbit IgG conjugated with IRDye^®^ 680RT (LI-COR Biosciences, Lincoln, NE, USA)) at RT for 1 h. The immunoreactive signal on the membranes was read by an Odyssey^®^ CLx Imaging System (LI-COR Biosciences). Quantification of Western blot band intensity was analyzed by the ImageJ software (version 1.51j8) (NIH, Bethesda, MD, USA).

### 2.7. Statistical Analysis

Each assay was conducted in at least 3 independent experiments. Analyses were carried out with the GraphPad Prism Software version 9.0.0 (121) (GraphPad Software Inc., San Diego, CA, USA). Data were represented as the mean ± SD and then analyzed using one-way ANOVA followed by Tukey’s multiple comparison test. *p* value < 0.05 was considered statistically significant.

## 3. Results

### 3.1. The Cell Viability/Proliferation of Human Endothelial Cells in Response to PA Treatment

**Figure 1:**
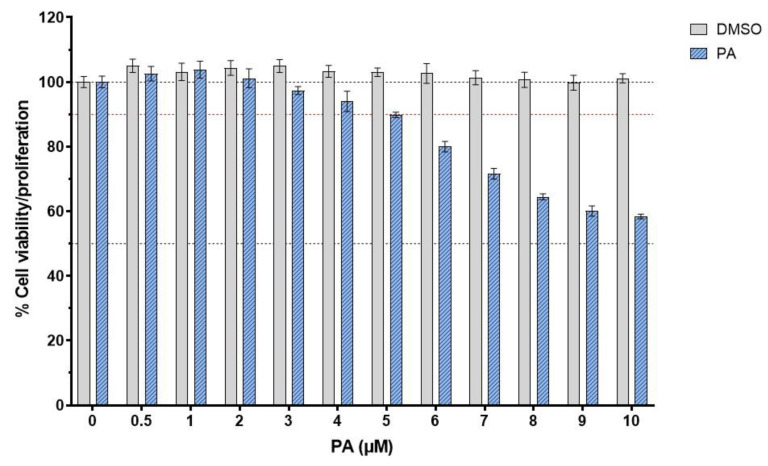
Effect of PA on the viability or proliferation of endothelial cells. Cells were treated with PA (at concentrations ranging from 0 to 10 μM) or DMSO (vehicle control) for 48 h prior to the cell viability test. Data are obtained from at least 3 individual experiments and presented as mean ± SD.

To elucidate the pharmacological activities of PA in suppressing endothelial inflammation, we needed to select the concentrations of this compound that did not kill human endothelial cells, HMEC-1. The viability/proliferation of the treated cells above 90% was considered safe for the cells. Results from the MTT assay demonstrated that DMSO (used as a vehicle control) showed no effect on the cell viability, whereas PA at concentrations above 4 μM was toxic to the cell because the cell viability/proliferation was lower than 90% (Figure 1). Therefore, we selected three concentrations (2, 3, and 4 μM) for all experiments.

### 3.2. The Level of IL-6 and MCP-1 in the Culture Media of TNF-α-Stimulated Endothelial Cells Treated with PA

**Figure 2:**
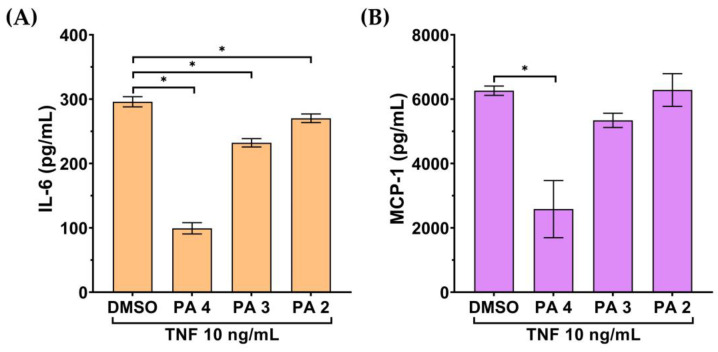
Effect of PA on secretion of IL-6 and MCP-1 in the TNF-α-induced endothelial cell culture media. The level of IL-6 (**A**) and MCP-1 (**B**) in the media of the PA-treated cells stimulated with 10 TNF-α for 24 h evaluated by ELISA. Data are presented as mean ± SD * *p* < 0.05 (compared with the DMSO-treated group).

On the basis that endothelial cells produce and secrete inflammatory cytokines and chemokines in response to inflammatory stimulation, we thus assayed the inhibitory action of PA on the secretion of inflammatory cytokine and chemokine in cells stimulated with TNF-α. The culture supernatants of the cells were tested for the level of IL-6 and MCP-1 by ELISA. As shown in Figure 2A,B, the culture supernatants collected from endothelial cells without any treatment had no detectable levels of IL-6 and MCP-1. IL-6 and MCP-1 were increased to around 300 pg/mL and 6000 pg/mL, respectively, after TNF-α treatment for 24 h. However, PA could significantly reduce the level of these two inflammatory markers in the culture supernatants of TNF-α-induced endothelial cells.

### 3.3. The Level of ICAM-1 and VCAM-1 on the Surface of TNF-α-Activated Endothelial Cells Treated with PA

**Figure 3:**
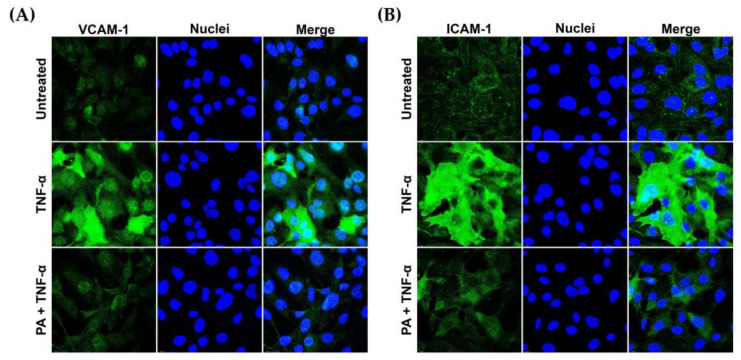
PA decreased TNF-α- induced expression of vascular adhesion molecules on endothelial cell surface. Endothelial cells (untreated, TNF-α-simulated, or TNF-α-simulated with the presence of PA at 4 μM for 24 h) were subject to immunofluorescence for detecting VCAM-1 (**A**) and ICAM-1 (**B**). Nuclei (blue) were visualized by DAPI staining. The micrographs were captured at 100× magnification.

Besides IL-6 and MCP-1, we tested the effect of PA on the presence of ICAM-1 and VCAM-1 on the surface of TNF-α-activated endothelial cells. Results from the immunofluorescence study demonstrated that stimulating endothelial cells with TNF-α for 24 h greatly increased the presence of VCAM-1 and ICAM-1 on the cell surface of the cells in comparison to that of the untreated cells. However, PA treatment decreased the production of VCAM-1 (Figure 3A) and ICAM-1 (Figure 3B) in response to TNF-α stimulation.

**Figure 4:**
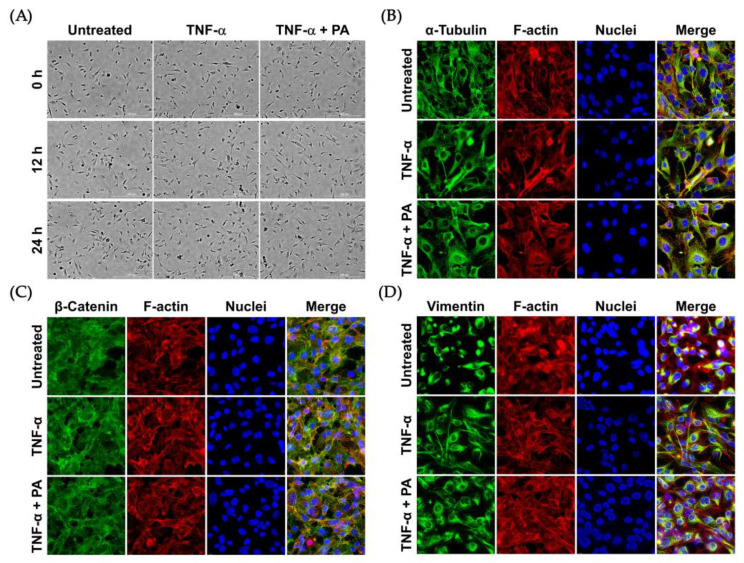
Effects of TNF-α and PA on the morphology and cytoskeletal arrangement of endothelial cells: (**A**) Phase-contrast microscopy visualizing endothelial cells without any treatment (Untreated), induced with TNF-α, or induced with TNF-α with the presence of 4 μM of PA at 0, 12, and 24 h (Scale bar = 300 μm). Immunofluorescence study detecting α-tubulin (**B**), β-catenin (**C**), and vimentin (**D**) in endothelial cells after treatment for 24 h. Cells were also stained for F-actin (red) and nuclei (blue). The micrographs (immunofluorescence study) were captured at 100× magnification.

Nevertheless, we observed that the morphology of endothelial cells was not drastically altered upon TNF-α or TNF-α + PA at different time points, from 0 to 24 h (Figure 4A). Consistently, TNF-α or TNF-α + PA did not affect the arrangement of filamentous actin, α-tubulin (Figure 4B), β-catenin (Figure 4C), and vimentin (Figure 4D).

### 3.4. Effects of PA on the Number of Monocytes Attached to the Surface of TNF-α-Activated Endothelial Cells

**Figure 5:**
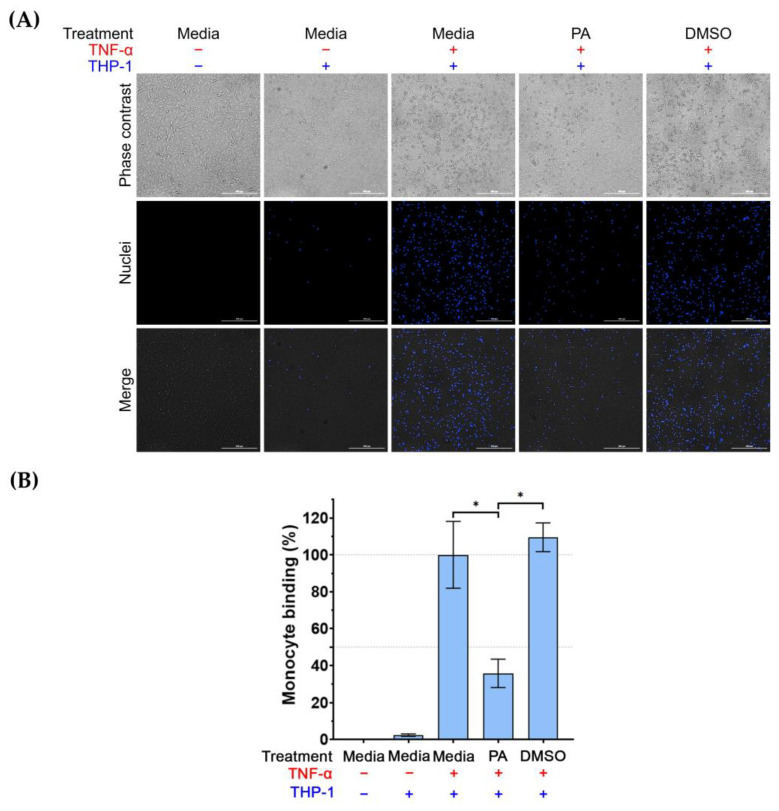
The effect of PA on THP-1 cell binding to TNF-α-activated endothelial cells. Adhesion of the labeled THP-1 monocytes to endothelial cells (untreated, induced with TNF-α, induced with TNF-α with the presence of PA at 4 μM, or induced with TNF-α with the presence of DMSO (vehicle control) for 24 h) was visualized and quantified by using a live-cell imager: (**A**) Phase-contrast microscopy showing THP-1 monocytes bound to endothelial monolayer, and fluorescent microscopy showing the nuclei (blue) of THP-1 monocytes. Scale bar = 300 μm. (**B**) Quantitative analysis of THP-1 cells bound to endothelial cells. Data are expressed as mean ± SD. * *p* < 0.05.

Since we observed that PA could decrease the presence of ICAM-1 and VCAM-1 on the surface of TNF-α-activated endothelial cells, it is possible that PA may reduce the binding of monocytes to TNF-α-activated endothelial cells. To prove this hypothesis, we performed an adhesion assay. Results (as shown in Figure 5A,B) revealed that the untreated endothelial cells without exposure to Hoechst 33342-labeled THP-1 cells showed an undetectable fluorescent signal. Stimulating endothelial cells with TNF-α dramatically increased THP-1 binding to endothelial cells. However, treatment of TNF-α-activated endothelial cells with PA (but not DMSO) significantly decreased THP-1 binding to endothelial cells.

### 3.5. Regulatory Activity of PA on the NF-κB Signal Transduction Pathway

**Figure 6:**
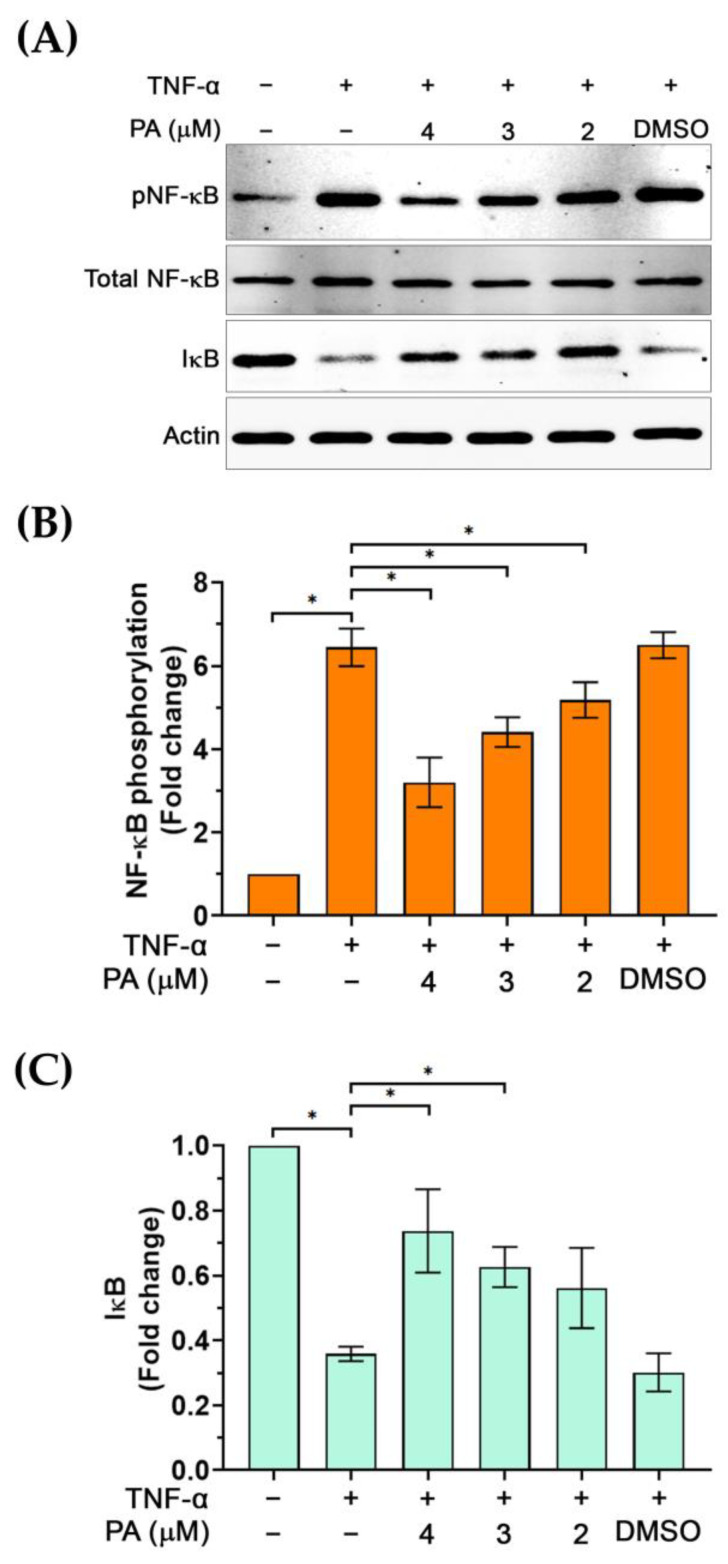
The effect of PA on TNF-α-activated phosphorylation of NF-κB (pNF-κB) and the degradation of IκB. (**A**) Western blot analysis showing the level of pNF-κB and IκB in cells without any treatment, TNF-α induction, and TNF-α induction with the presence of PA, or TNF-α induction with the presence of DMSO (vehicle control). (**B**) Densitometry of pNF-κB immunoreactive bands from cells without any treatment, TNF-α induction, and TNF-α induction with the presence of PA, or TNF-α induction with the presence of DMSO. (**C**) Densitometry of IκB immunoreactive bands from cells without any treatment, TNF-α induction, and TNF-α induction with the presence of PA, or TNF-α induction with the presence of DMSO. * *p* < 0.05. Original images of (**A**) can be found in Supplementary Materials.

Based on our observations that PA reduced the level of endothelial activation markers, which are downstream targets of the NF-κB signaling pathway, we thus investigated whether PA possesses inhibitory activity on this signaling pathway. We monitored the phosphorylation status of NF-κB (pNF-κB) and the level of IκB in TNF-α-activated endothelial cells with or without the presence of PA. Data showed that TNF-α stimulation significantly induced phosphorylation of NF-κB to approximately 6-fold compared to its basal level, and the level of pNF-κB in the TNF-α-activated cells treated with DMSO (vehicle control) was not reduced (Figure 6A,B). However, PA at 4, 3, and 2 μM significantly reduced phosphorylation of NF-κB to about 3, 4, and 5-fold, respectively (Figure 6A,B). Notably, the expression of total NF-κB was not changed by PA treatment. Consistently, cells exposed to TNF-α exhibited a decreased level of IκB (to approximately 0.3-fold in comparison to that of the untreated cells); however, PA at 4 and 3 μM significantly inhibited this TNF-α-activated degradation of IκB (Figure 6A,C).

**Figure 7:**
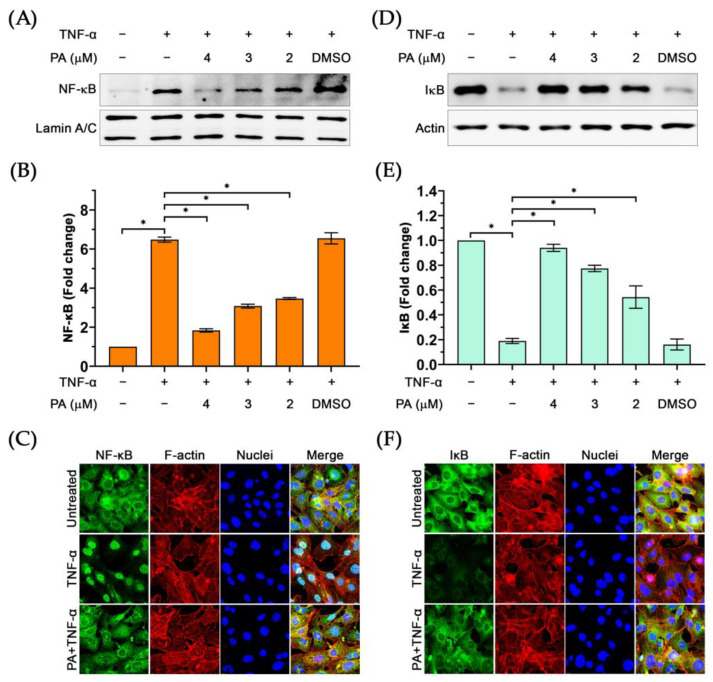
The effect of PA on inhibiting TNF-α-activated nuclear relocalization of NF-κB and IκB degradation: (**A**) The presence of NF-κB in the nuclear extract of TNF-α-activated endothelial cells with or without the presence of PA. (**B**) Densitometric analysis of NF-κB immunoreactive bands from the Western blot of the nuclear extracts. (**C**) Immunofluorescence study visualizing relocalization of NF-κB into the nucleus of TNF-α-activated endothelial cells with or without the presence of PA. (**D**) Western blot analysis detecting IκB in the cytoplasmic extracts of TNF-α-activated endothelial cells with or without the presence of PA. (**E**) Densitometric analysis of IκB immunoreactive bands from the Western blot of the cytoplasmic extracts. (**F**) Immunofluorescence study visualizing the existence of IκB in the cytosol of TNF-α-activated endothelial cells with or without the presence of PA. * *p* < 0.05. The micrographs were captured at 100× magnification. Original images of (**A**,**D**) can be found in Supplementary Materials.

The observation that PA reduced the phosphorylation of NF-κB upon stimulation with TNF-α posed the possibility that PA may block the translocation of NF-κB into the nucleus. Thus, we performed nuclear and cytoplasmic extracts of cells upon PA treatment and TNF-α stimulation. Results from Western blot analysis demonstrated that the presence of NF-κB in the nuclear extract of TNF-α-stimulated cells was increased (about 6-fold) in comparison to that of the untreated cells (Figure 7A,B). Pretreatment of cells with PA at 4, 3, and 2 μM for 3 h prior to TNF-α stimulation for 30 min caused a significant reduction in NF-κB level in the nuclear extract of the cells without any effect on the level of nuclear Lamin A/C (a marker for the nuclear compartment) (Figure 7A,B). These results were supported by data from an immunofluorescence study where we visualized that PA strongly inhibited the relocalization of NF-κB into the cell nucleus (Figure 7C). Additionally, we detected the existence of IκB in the cytoplasmic extract of the cells upon PA treatment and TNF-α stimulation and found that PA at all concentrations could significantly prevent TNF-α-induced IκB degradation (Figure 7D,E). Also, data from immunostaining clearly indicated that PA treatment retained IκB in the cytosolic compartment of the TNF-α-stimulated endothelial cells (Figure 7F).

**Figure 8:**
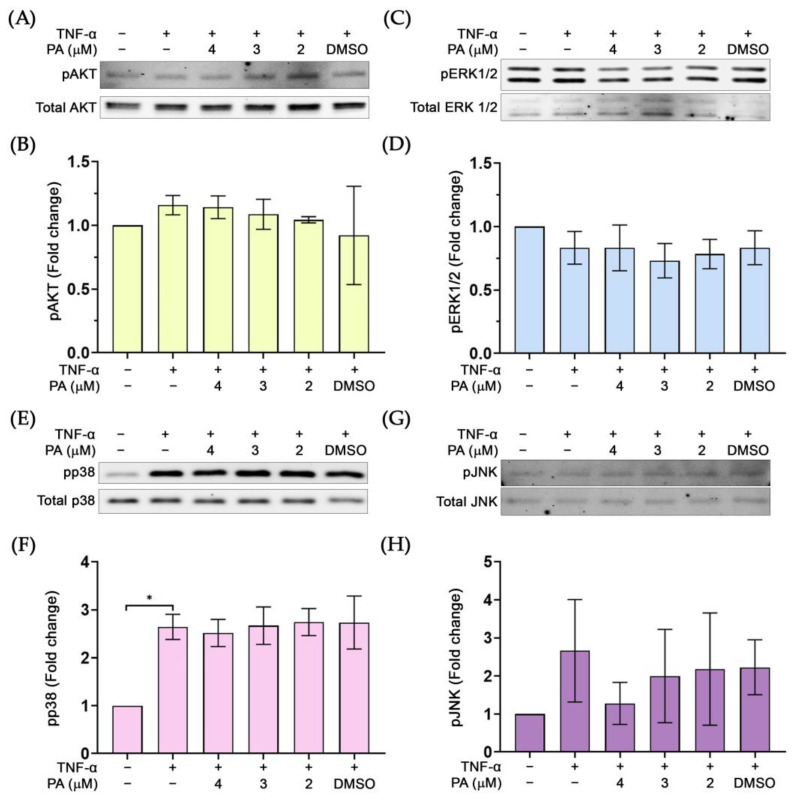
The effect of PA on the phosphorylation status of important molecular markers for growth and survival: (**A**) Western blot determining the level of pAKT. (**B**) Quantitative analysis of pAKT immunoreactive bands. (**C**) Western blot determining the level of pERK1/2. (**D**) Quantitative analysis of pERK1/2 immunoreactive bands. (**E**) Western blot analysis detecting pp38. (**F**) Quantitative analysis of pp38 immunoreactive bands. (**G**) Western blot analysis detecting pJNK. (**H**) Quantitative analysis of pJNK immunoreactive bands. * *p* < 0.05. Original images of (**A**,**C**,**E**,**G**) can be found in Supplementary Materials.

We further examined whether PA possesses the inhibitory activity on other molecular signal transduction pathways including the growth and survival signalings. Results revealed that PA did not reduce phosphorylation of AKT (Figure 8A,B), ERK1/2 (Figure 8C,D), p38 (Figure 8E,F), and JNK (Figure 8G,H).

## 4. Discussion

Uncontrollable inflammation and dysfunction of endothelium contribute to the development of vascular disease including atherosclerosis [16]. One of the well-known inflammatory stimuli is TNF-α, which activates endothelial cells. The current study explored the pharmacological effect of PA on reducing crucial markers of endothelial inflammation and activation in response to TNF-α treatment as well as its underlying mechanism of action.

As it is known that endothelial cells are major targets of extracellular stimuli including TNF-α, which activates the generation of pro-inflammatory cytokines [17,18], we thus examined the effect of PA on the level of key pro-inflammatory molecules in the media of endothelial cells treated with TNF-α. We found that PA could reduce the level of IL-6 in the culture media collected from TNF-α-exposed endothelial cells. In line with our observations, there has been a report demonstrating that PA suppressed IL-6 (along with other cytokines) expression in mouse microglial cells induced with LPS [13]. Additionally, we found that PA reduced the level of MCP-1, a chemokine that proved to be involved in the pathogenesis of several diseases including atherosclerosis [19].

The observation that PA could suppress IL-6 and MCP-1 suggests that PA may have an inhibitory effect on endothelial inflammation. Besides cytokines and chemokines, ICAM-1 and VCAM-1 have been identified as markers of endothelial inflammation and dysfunction upon exposure to different stimuli [20,21,22] including TNF-α [23,24]. Importantly, an increase in the expression of these adhesion molecules on the endothelial cell surface is linked to a high incidence of coronary heart disease [25]. Therefore, we evaluated the effect of PA on the expression of these key adhesion molecules and found that PA could markedly reduce the influence of TNF-α on stimulating the production of ICAM-1 and VCAM-1 on the endothelial surface without any effect on the cell morphology or the rearrangement of major cytoskeletal proteins including filamentous actin, α-tubulin, β-catenin, and vimentin. ICAM-1 and VCAM-1 expressed on the surface of endothelial cells facilitate tight binding between immune cells and endothelial cells, which consequently results in immune cell extravasation along with the secretion of inflammatory molecules and lipid overload, which eventually contributes to atherosclerotic plaques [26,27].

The reduction in these adhesion molecules by PA may result in a reduction in monocyte recruitment. To prove this hypothesis, we observed the effect of PA on lowering the number of monocytes adhered to TNF-α-stimulated endothelial cells. Data revealed that PA significantly decreased the number of monocytes on the surface of TNF-α-activated endothelial cells. Since impeding immune cell adhesion to endothelial cells is a characteristic of early atherosclerosis [28,29], it is possible that PA may be able to prevent the generation of atherosclerosis, at least in part, through inhibiting cytokine and chemokine production, reducing surface expression of ICAM-1 and VCAM-1, and lowering the number of monocytes on TNF-α-activated endothelial cells.

We further identified the possible molecular mechanism of action of PA by focusing on the NF-κB pathway because it is a major mechanism contributing to endothelial activation in response to TNF-α stimulation [30,31]. Specifically, NF-κB activation is responsible for the release of IL-6, MCP-1, ICAM-1, and VCAM-1 [32,33]. We demonstrated that PA inhibited phosphorylation of NF-κB while suppressing the degradation of IκB upon TNF-α stimulation, which subsequently prevented nuclear translocation of NF-κB. Retention of IκB in the cytosol while prevention of relocalization of NF-κB into the nucleus of the cell indicates that PA suppresses NF-κB activation, and that explains its inhibitory actions on the production of endothelial activation markers upon TNF-α stimulation. However, PA may also exert its regulatory activity on other important signaling. The PI3K/AKT pathway has been well established to play a crucial role in regulating cell survival, proliferation, and metabolism, and dysregulation of this pathway can lead to the development of certain diseases [34,35]. In particular, it has been reported that H_2_O_2_ generated reactive oxygen species (ROS) which subsequently activate the phosphorylation of AKT and ERK1/2 kinases in endothelial cells, and glucagon-like peptide-1 (GLP-1) attenuated ROS production accompanied with reducing AKT and ERK1/2 phosphorylation to reduce endothelial dysfunction and autophagy [36]. Based on these findings, we explored the effect of PA on the activation status of AKT and ERK1/2 in TNF-α-stimulated endothelial cells. We demonstrated that in response to TNF-α, AKT and ERK1/2 phosphorylation was not induced, and PA did not have any effect on the basal level of phosphorylated AKT and ERK1/2. These results suggest that suppression of the ROS-induced AKT and ERK1/2 signaling pathway may not be the major mechanism of PA, even though PA has been shown to possess antioxidant activity [37]. Although it has been reported that the expression of cell adhesion molecules in endothelial cells in response to TNF-α stimulation was mainly regulated by NF-κB, but not p38 MAP kinase [38], we still explored the effect of PA on p38 activation. Data showed that TNF-α strongly stimulated p38 phosphorylation, but PA did not reduce p38 phosphorylation. In addition, consistent with a previous report where it showed that TNF-α had no stimulating effect on JNK [39], we observed that TNF-α did not activate JNK, and PA did not interfere with the basal phosphorylation status of JNK. Altogether, these results suggest that the activity of PA on the TNF-α-activated NF-κB signaling is relatively specific. There has been a previous study reporting that PA exhibited anti-inflammatory activity in microglia stimulated with LPS by reducing nitric oxide and cytokine production via blocking NF-κB activation [13]. In periodontitis-induced inflammation model, PA inhibited NF-κB expression in human gingival fibroblast-1 stimulated with LPS [14]. Although the potential molecular mechanism of action of PA on suppressing TNF-α-stimulated endothelial activation was clearly defined to be through inhibiting the NF-κB signaling, its possible regulatory activities in other inflammatory mechanisms remain to be further elucidated, especially the possible modulatory role on the IL-6/JAK/STAT signaling axis [40] which is involved in the cytokine response in endothelial cells [41,42].

**Figure 9:**
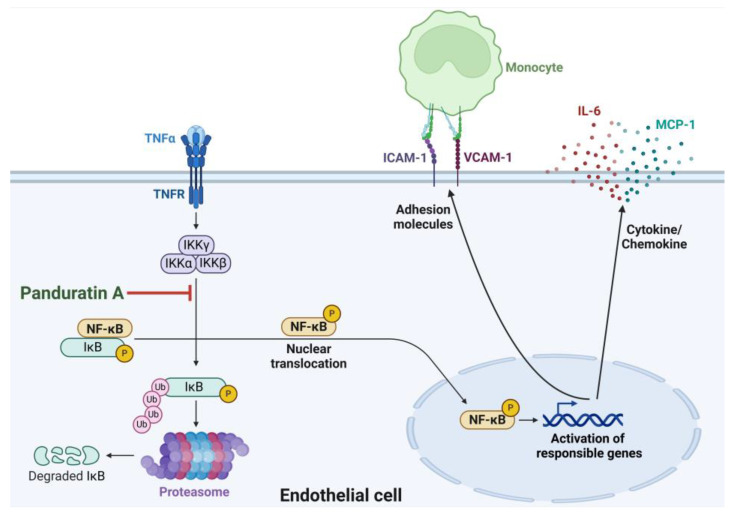
The proposed model for the mechanism of PA in reducing TNF-α-stimulated endothelial activation and monocyte adhesion. This figure was created with an approval from BioRender.com (accessed on 28 November 2024).

In summary, our study (as illustrated in Figure 9) demonstrated that PA effectively inhibited IL-6 and MCP-1 secretion, ICAM-1 and VCAM-1 production, and adhesion of monocytes on endothelial cells stimulated with TNF-α through suppression of NF-κB activation. Our findings provided accumulated evidence about the mechanisms of action of panduratin A underling its inhibitory effects on the key markers of endothelial activation, which suggests that panduratin A may be beneficial for preventing vascular inflammation and early atherosclerotic lesions. Since studies associated with the potential pharmacological effect of PA have been accumulated, there have been reports about the safety and the pharmacokinetics of PA in different animal models [43,44,45] which may assist in developing PA or PA-containing plant extract for preventing diseases associated with imbalanced endothelial activation.

## 5. Conclusions

The current study revealed that panduratin A exhibits inhibitory activities against the effects of TNF-α. The compound suppressed the production of IL-6, MCP-1, ICAM-1, and VCAM-1, which consequently inhibited monocyte adhesion to endothelial cells. We identified that these properties of panduratin A are mediated, at least in part, through the blockade of the NF-κB pathway. This study provides evidence supporting that panduratin A may be an interesting candidate for preventing endothelial inflammation and dysfunction which are critical factors contributing to the development of atherosclerosis.

## Data Availability

All data, tables, and figures are original and are available in this article.

## Supplementary Materials

The following supporting information can be downloaded at: https://www.mdpi.com/article/10.3390/biom15010034/s1, Original images of Figure 6A, Figure 7A,D and Figure 8A,C,E,G can be found in Supplementary Materials.

## References

[1] Fan J., Watanabe T. (2022). Atherosclerosis: Known and unknown. Pathol. Int..

[2] Theofilis P., Sagris M., Oikonomou E., Antonopoulos A.S., Siasos G., Tsioufis C., Tousoulis D. (2021). Inflammatory Mechanisms Contributing to Endothelial Dysfunction. Biomedicines.

[3] Pober J.S. (2002). Endothelial activation: Intracellular signaling pathways. Arthritis Res..

[4] Sehnert B., Burkhardt H., Wessels J.T., Schroder A., May M.J., Vestweber D., Zwerina J., Warnatz K., Nimmerjahn F., Schett G. (2013). NF-kappaB inhibitor targeted to activated endothelium demonstrates a critical role of endothelial NF-kappaB in immune-mediated diseases. Proc. Natl. Acad. Sci. USA.

[5] Ley K., Laudanna C., Cybulsky M.I., Nourshargh S. (2007). Getting to the site of inflammation: The leukocyte adhesion cascade updated. Nat. Rev. Immunol..

[6] Alfaidi M., Wilson H., Daigneault M., Burnett A., Ridger V., Chamberlain J., Francis S. (2015). Neutrophil elastase promotes interleukin-1beta secretion from human coronary endothelium. J. Biol. Chem..

[7] Ridker P.M., Rane M. (2021). Interleukin-6 Signaling and Anti-Interleukin-6 Therapeutics in Cardiovascular Disease. Circ. Res..

[8] Zhou Z., Albarqouni L., Breslin M., Curtis A.J., Nelson M. (2017). Statin-associated muscle symptoms (SAMS) in primary prevention for cardiovascular disease in older adults: A protocol for a systematic review and meta-analysis of randomised controlled trials. BMJ Open.

[9] Ward N.C., Watts G.F., Eckel R.H. (2019). Statin Toxicity. Circ. Res..

[10] Danelich I.M., Wright S.S., Lose J.M., Tefft B.J., Cicci J.D., Reed B.N. (2015). Safety of nonsteroidal antiinflammatory drugs in patients with cardiovascular disease. Pharmacotherapy.

[11] Vasudevan A., Ip F., Liew D., van Langenberg D.R. (2020). The Cost-effectiveness of Initial Immunomodulators or Infliximab Using Modern Optimization Strategies for Crohn’s Disease in the Biosimilar Era. Inflamm. Bowel Dis..

[12] Eng-Chong T., Yean-Kee L., Chin-Fei C., Choon-Han H., Sher-Ming W., Li-Ping C.T., Gen-Teck F., Khalid N., Abd Rahman N., Karsani S.A. (2012). Boesenbergia rotunda: From Ethnomedicine to Drug Discovery. Evid. Based Complement. Altern. Med..

[13] Jamornwan S., Chokpanuwat T., Uppakara K., Soodvilai S., Saengsawang W. (2022). Anti-Inflammatory Activity of Panduratin A against LPS-Induced Microglial Activation. Biomedicines.

[14] Kim H., Kim M.B., Kim C., Hwang J.K. (2018). Inhibitory Effects of Panduratin A on Periodontitis-Induced Inflammation and Osteoclastogenesis through Inhibition of MAPK Pathways In Vitro. J. Microbiol. Biotechnol..

[15] Cheah S.C., Appleton D.R., Lee S.T., Lam M.L., Hadi A.H., Mustafa M.R. (2011). Panduratin A inhibits the growth of A549 cells through induction of apoptosis and inhibition of NF-kappaB translocation. Molecules.

[16] Cines D.B., Pollak E.S., Buck C.A., Loscalzo J., Zimmerman G.A., McEver R.P., Pober J.S., Wick T.M., Konkle B.A., Schwartz B.S. (1998). Endothelial cells in physiology and in the pathophysiology of vascular disorders. Blood.

[17] Jirik F.R., Podor T.J., Hirano T., Kishimoto T., Loskutoff D.J., Carson D.A., Lotz M. (1989). Bacterial lipopolysaccharide and inflammatory mediators augment IL-6 secretion by human endothelial cells. J. Immunol..

[18] Nawroth P.P., Bank I., Handley D., Cassimeris J., Chess L., Stern D. (1986). Tumor necrosis factor/cachectin interacts with endothelial cell receptors to induce release of interleukin 1. J. Exp. Med..

[19] Xia M., Sui Z. (2009). Recent developments in CCR2 antagonists. Expert. Opin. Ther. Pat..

[20] Little P.J., Askew C.D., Xu S., Kamato D. (2021). Endothelial Dysfunction and Cardiovascular Disease: History and Analysis of the Clinical Utility of the Relationship. Biomedicines.

[21] Nafisa A., Gray S.G., Cao Y., Wang T., Xu S., Wattoo F.H., Barras M., Cohen N., Kamato D., Little P.J. (2018). Endothelial function and dysfunction: Impact of metformin. Pharmacol. Ther..

[22] Dri E., Lampas E., Lazaros G., Lazarou E., Theofilis P., Tsioufis C., Tousoulis D. (2023). Inflammatory Mediators of Endothelial Dysfunction. Life.

[23] Hou H.F., Yuan N., Guo Q., Sun T., Li C., Liu J.B., Li Q.W., Jiang B.F. (2015). Citreoviridin Enhances Atherogenesis in Hypercholesterolemic ApoE-Deficient Mice via Upregulating Inflammation and Endothelial Dysfunction. PLoS ONE.

[24] Koo H.J., Sohn E.H., Pyo S., Woo H.G., Park D.W., Ham Y.M., Jang S.A., Park S.Y., Kang S.C. (2015). An ethanol root extract of Cynanchum wilfordii containing acetophenones suppresses the expression of VCAM-1 and ICAM-1 in TNF-alpha-stimulated human aortic smooth muscle cells through the NF-kappaB pathway. Int. J. Mol. Med..

[25] Blankenberg S., Rupprecht H.J., Bickel C., Peetz D., Hafner G., Tiret L., Meyer J. (2001). Circulating cell adhesion molecules and death in patients with coronary artery disease. Circulation.

[26] Blankenberg S., Barbaux S., Tiret L. (2003). Adhesion molecules and atherosclerosis. Atherosclerosis.

[27] Lu Y., Zhu X., Liang G.X., Cui R.R., Liu Y., Wu S.S., Liang Q.H., Liu G.Y., Jiang Y., Liao X.B. (2012). Apelin-APJ induces ICAM-1, VCAM-1 and MCP-1 expression via NF-kappaB/JNK signal pathway in human umbilical vein endothelial cells. Amino Acids.

[28] Libby P. (2012). Inflammation in atherosclerosis. Arterioscler. Thromb. Vasc. Biol..

[29] Hansson G.K. (2005). Inflammation, atherosclerosis, and coronary artery disease. N. Engl. J. Med..

[30] Gustin J.A., Pincheira R., Mayo L.D., Ozes O.N., Kessler K.M., Baerwald M.R., Korgaonkar C.K., Donner D.B. (2004). Tumor necrosis factor activates CRE-binding protein through a p38 MAPK/MSK1 signaling pathway in endothelial cells. Am. J. Physiol. Cell Physiol..

[31] Monaco C., Paleolog E. (2004). Nuclear factor kappaB: A potential therapeutic target in atherosclerosis and thrombosis. Cardiovasc. Res..

[32] Anderson M.T., Staal F.J., Gitler C., Herzenberg L.A., Herzenberg L.A. (1994). Separation of oxidant-initiated and redox-regulated steps in the NF-kappa B signal transduction pathway. Proc. Natl. Acad. Sci. USA.

[33] Collins T., Read M.A., Neish A.S., Whitley M.Z., Thanos D., Maniatis T. (1995). Transcriptional regulation of endothelial cell adhesion molecules: NF-kappa B and cytokine-inducible enhancers. FASEB J..

[34] Hers I., Vincent E.E., Tavare J.M. (2011). Akt signalling in health and disease. Cell Signal.

[35] Altomare D.A., Khaled A.R. (2012). Homeostasis and the importance for a balance between AKT/mTOR activity and intracellular signaling. Curr. Med. Chem..

[36] Cai X., She M., Xu M., Chen H., Li J., Chen X., Zheng D., Liu J., Chen S., Zhu J. (2018). GLP-1 treatment protects endothelial cells from oxidative stress-induced autophagy and endothelial dysfunction. Int. J. Biol. Sci..

[37] Sohn J.H., Han K.L., Lee S.H., Hwang J.K. (2005). Protective effects of panduratin A against oxidative damage of tert-butylhydroperoxide in human HepG2 cells. Biol. Pharm. Bull..

[38] Rajan S., Ye J., Bai S., Huang F., Guo Y.L. (2008). NF-kappaB, but not p38 MAP kinase, is required for TNF-alpha-induced expression of cell adhesion molecules in endothelial cells. J. Cell Biochem..

[39] Li J.M., Fan L.M., Christie M.R., Shah A.M. (2005). Acute tumor necrosis factor alpha signaling via NADPH oxidase in microvascular endothelial cells: Role of p47phox phosphorylation and binding to TRAF4. Mol. Cell Biol..

[40] Kang S., Narazaki M., Metwally H., Kishimoto T. (2020). Correction: Historical overview of the interleukin-6 family cytokine. J. Exp. Med..

[41] Kang S., Tanaka T., Inoue H., Ono C., Hashimoto S., Kioi Y., Matsumoto H., Matsuura H., Matsubara T., Shimizu K. (2020). IL-6 trans-signaling induces plasminogen activator inhibitor-1 from vascular endothelial cells in cytokine release syndrome. Proc. Natl. Acad. Sci. USA.

[42] Kang S., Kishimoto T. (2021). Interplay between interleukin-6 signaling and the vascular endothelium in cytokine storms. Exp. Mol. Med..

[43] Kongratanapasert T., Boonyarattanasoonthorn T., Supannapan K., Hongeng S., Khemawoot P. (2024). Oral Bioavailability, Tissue Distribution, Metabolism, and Excretion of Panduratin A from Boesenbergia rotunda Extract in Healthy Rats. Drug Des. Devel. Ther..

[44] Boonyarattanasoonthorn T., Kongratanapasert T., Jiso A., Techapichetvanich P., Nuengchamnong N., Supannapan K., Kijtawornrat A., Khemawoot P. (2023). Absolute oral bioavailability and possible metabolic pathway of panduratin A from Boesenbergia rotunda extract in beagle dogs. Pharm. Biol..

[45] Won J., Noh K., Hwang J.K., Shin B.S., Kang W. (2021). Pharmacokinetics of panduratin A following oral administration of a Boesenbergia pandurata extract to rats. J. Food Drug Anal..

