# Supplementary figures and images for: Panduratin A Inhibits TNF Alpha-Stimulated Endothelial Cell Activation Through Suppressing the NF-κB Pathway
Source: Biomolecules. 2024 Dec 30;15(1):34. doi: 10.3390/biom15010034 (PMC11762725; doi:10.3390/biom15010034)

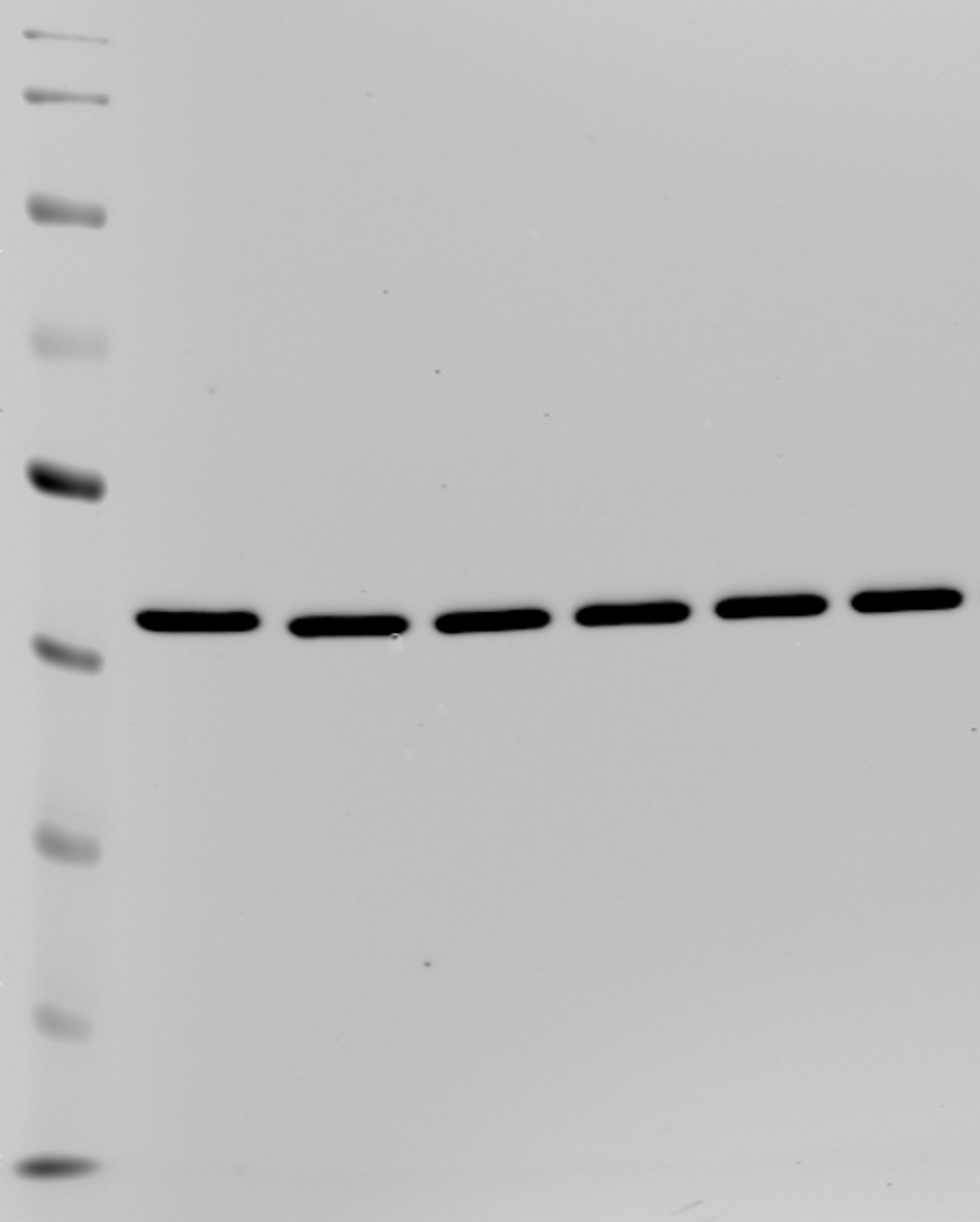

Supplement: Supplementary file 1 [file biomolecules-15-00034-s001.zip › Western blot raw data/Figure 6_Actin_N1.jpg]

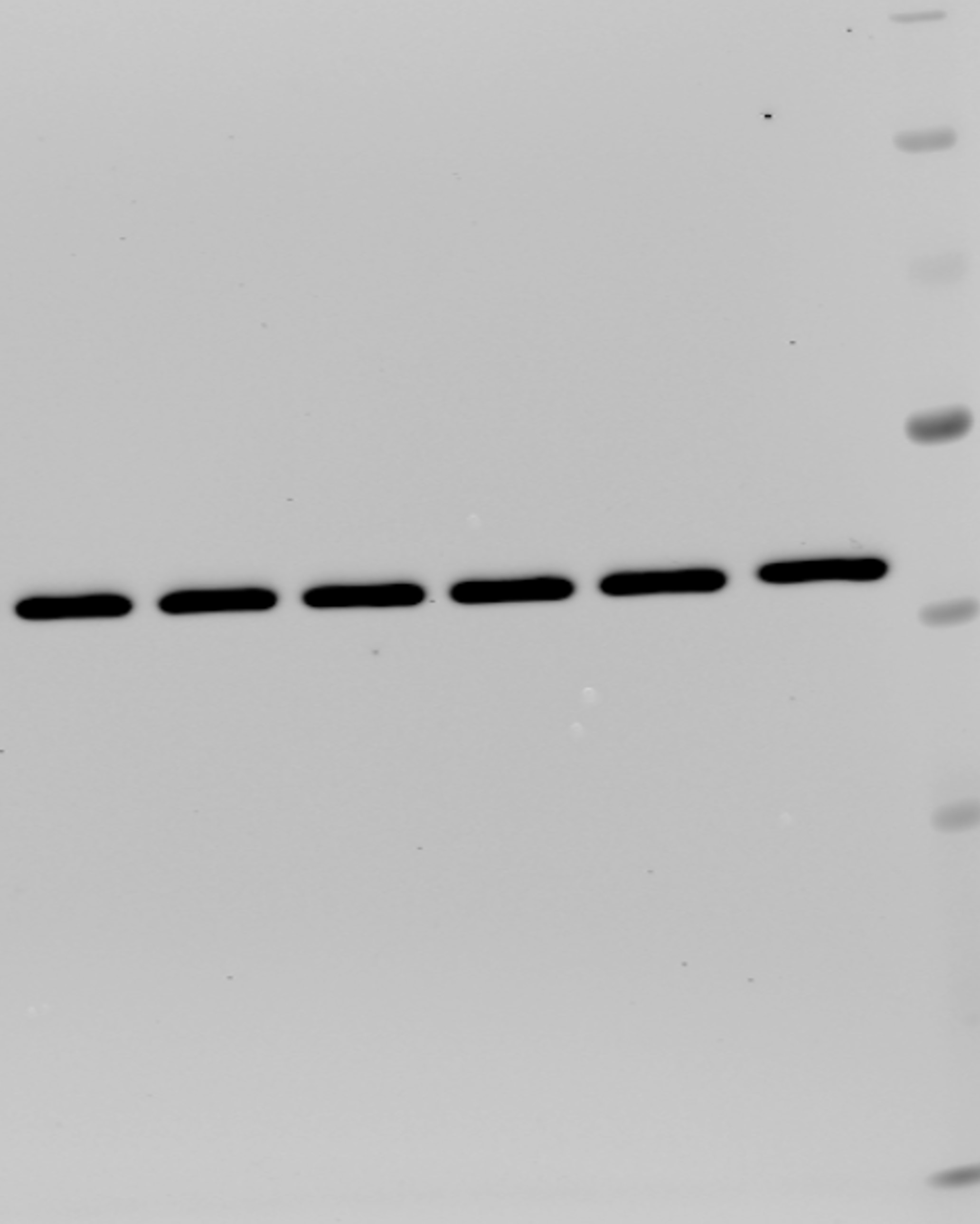

Supplement: Supplementary file 1 [file biomolecules-15-00034-s001.zip › Western blot raw data/Figure 6_Actin_N2.jpg]

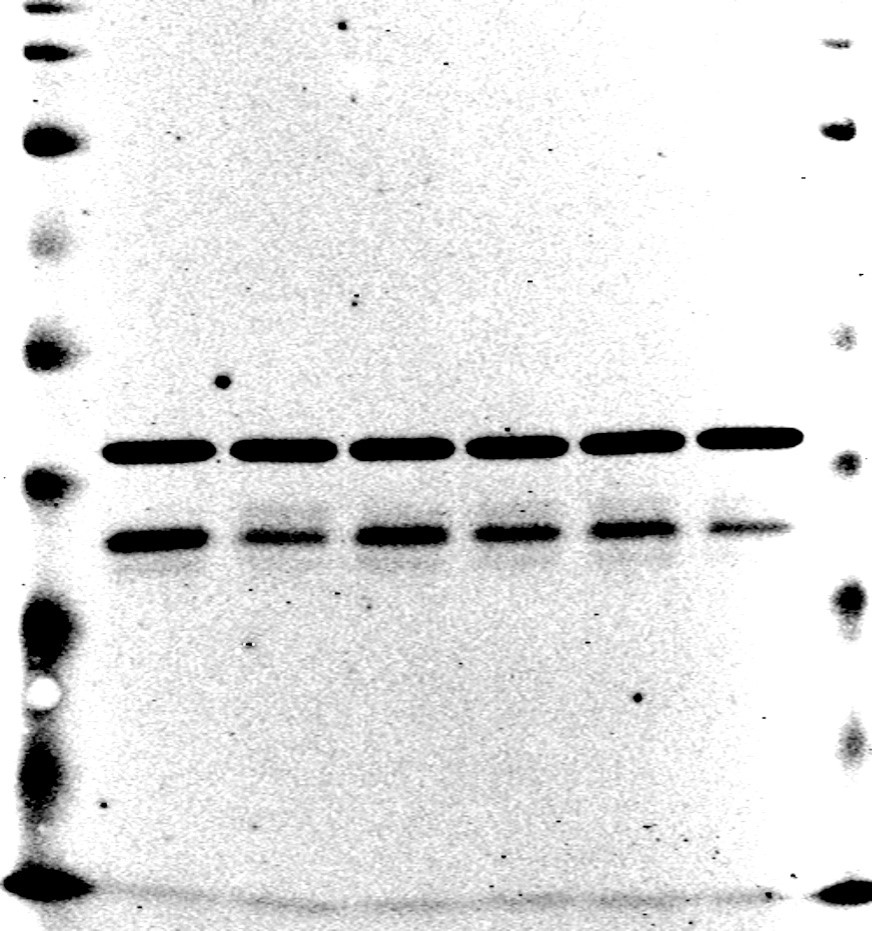

Supplement: Supplementary file 1 [file biomolecules-15-00034-s001.zip › Western blot raw data/Figure 6_Actin_N3.jpg]

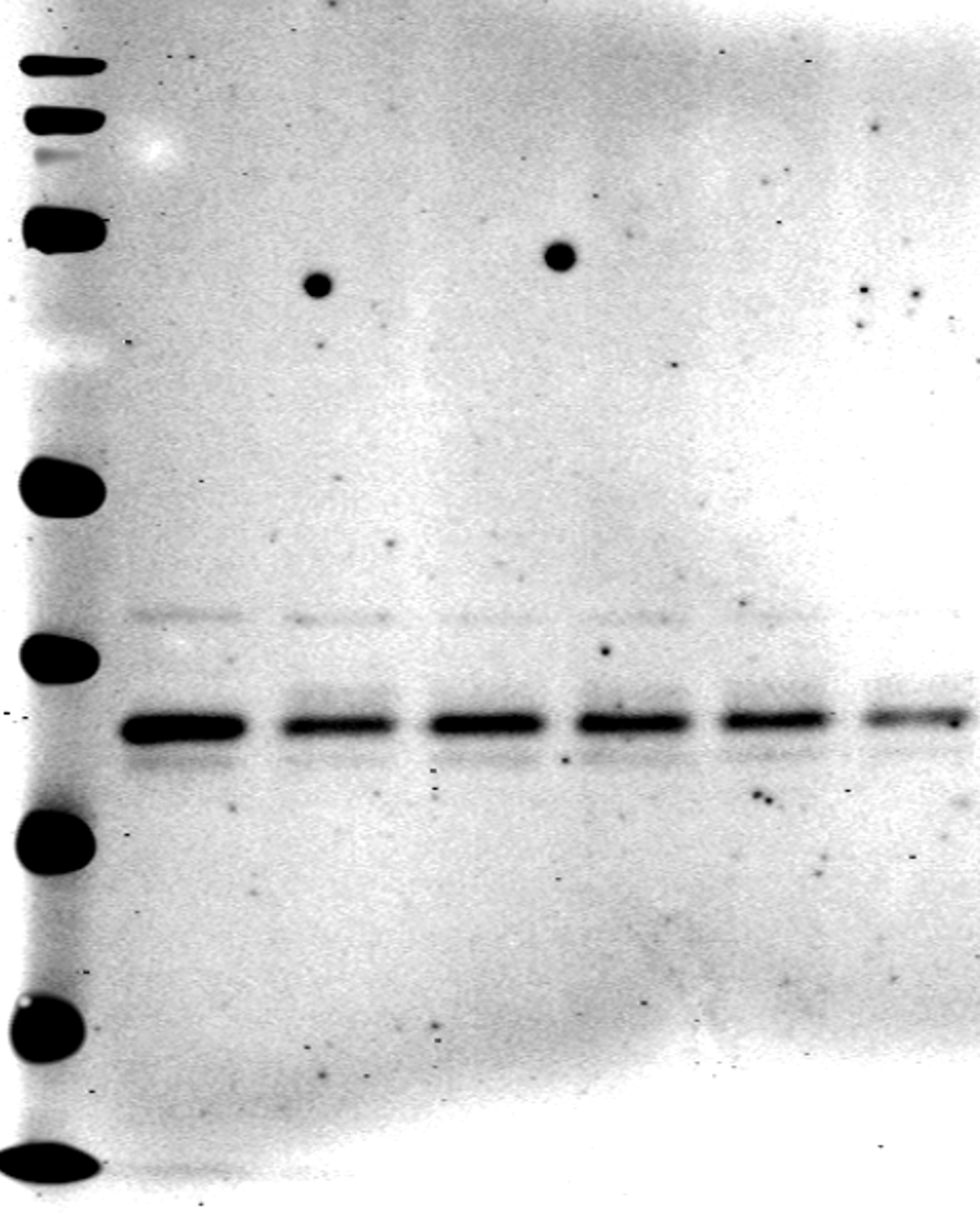

Supplement: Supplementary file 1 [file biomolecules-15-00034-s001.zip › Western blot raw data/Figure 6_IkB_N1.jpg]

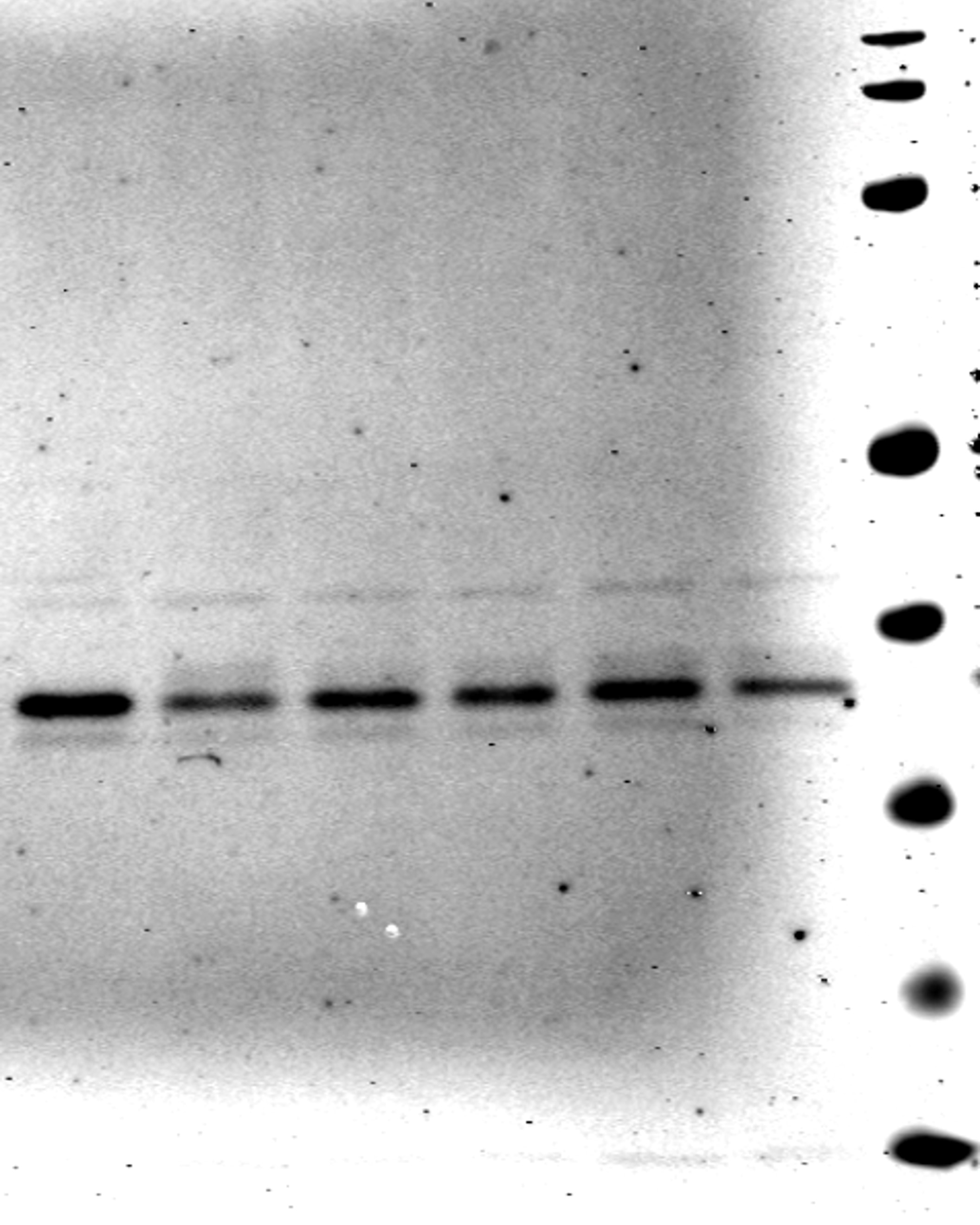

Supplement: Supplementary file 1 [file biomolecules-15-00034-s001.zip › Western blot raw data/Figure 6_IkB_N2.jpg]

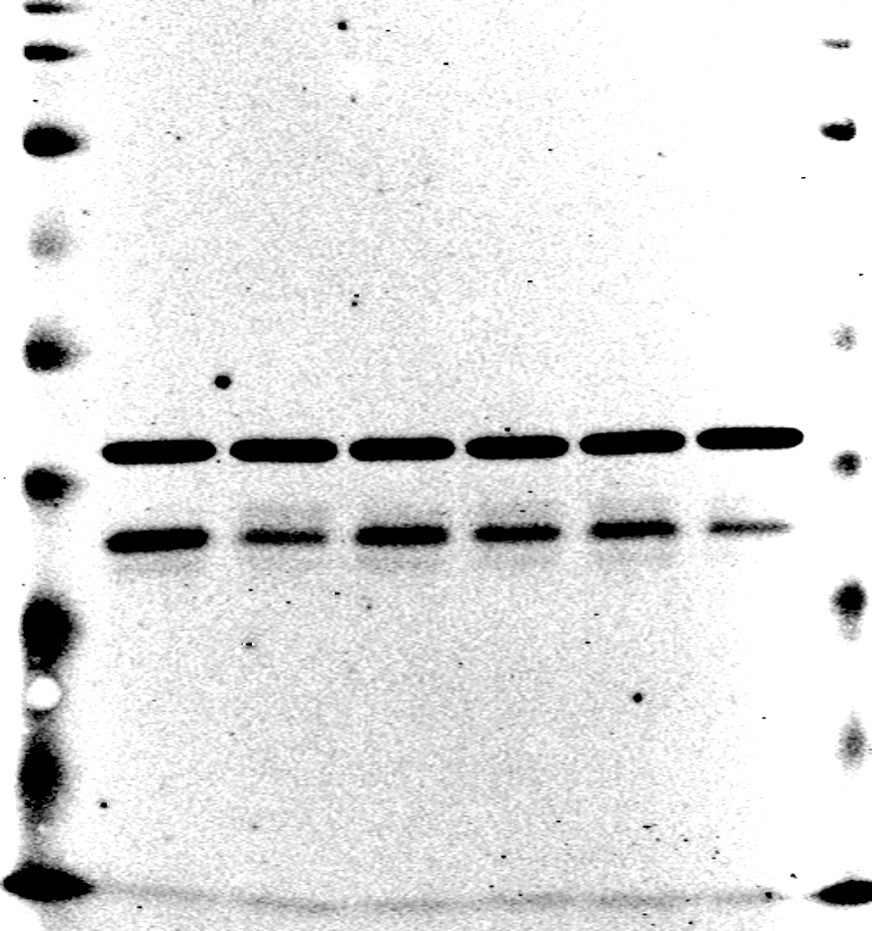

Supplement: Supplementary file 1 [file biomolecules-15-00034-s001.zip › Western blot raw data/Figure 6_IkB_N3.jpg]

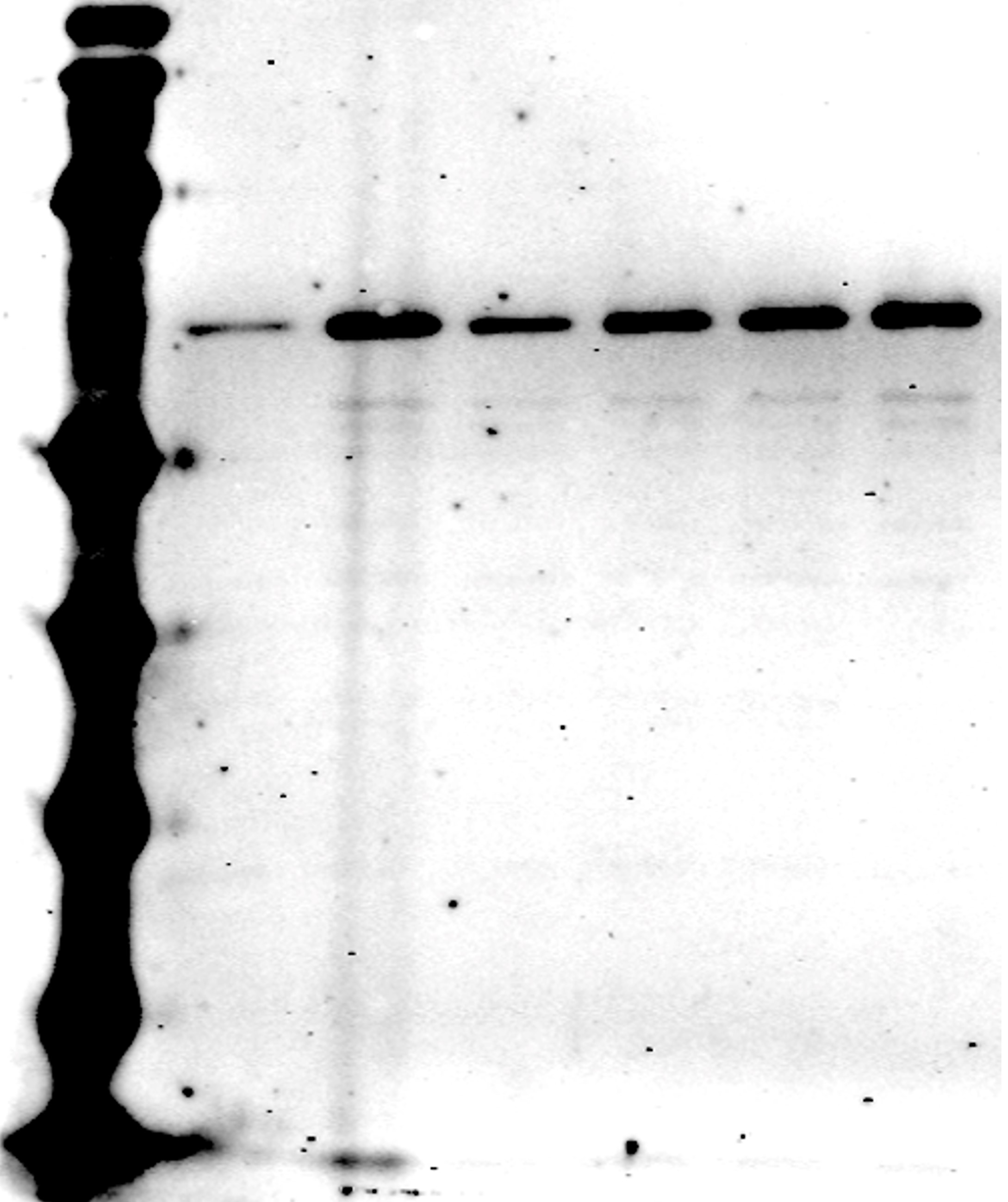

Supplement: Supplementary file 1 [file biomolecules-15-00034-s001.zip › Western blot raw data/Figure 6_pNF-kB_N1.jpg]

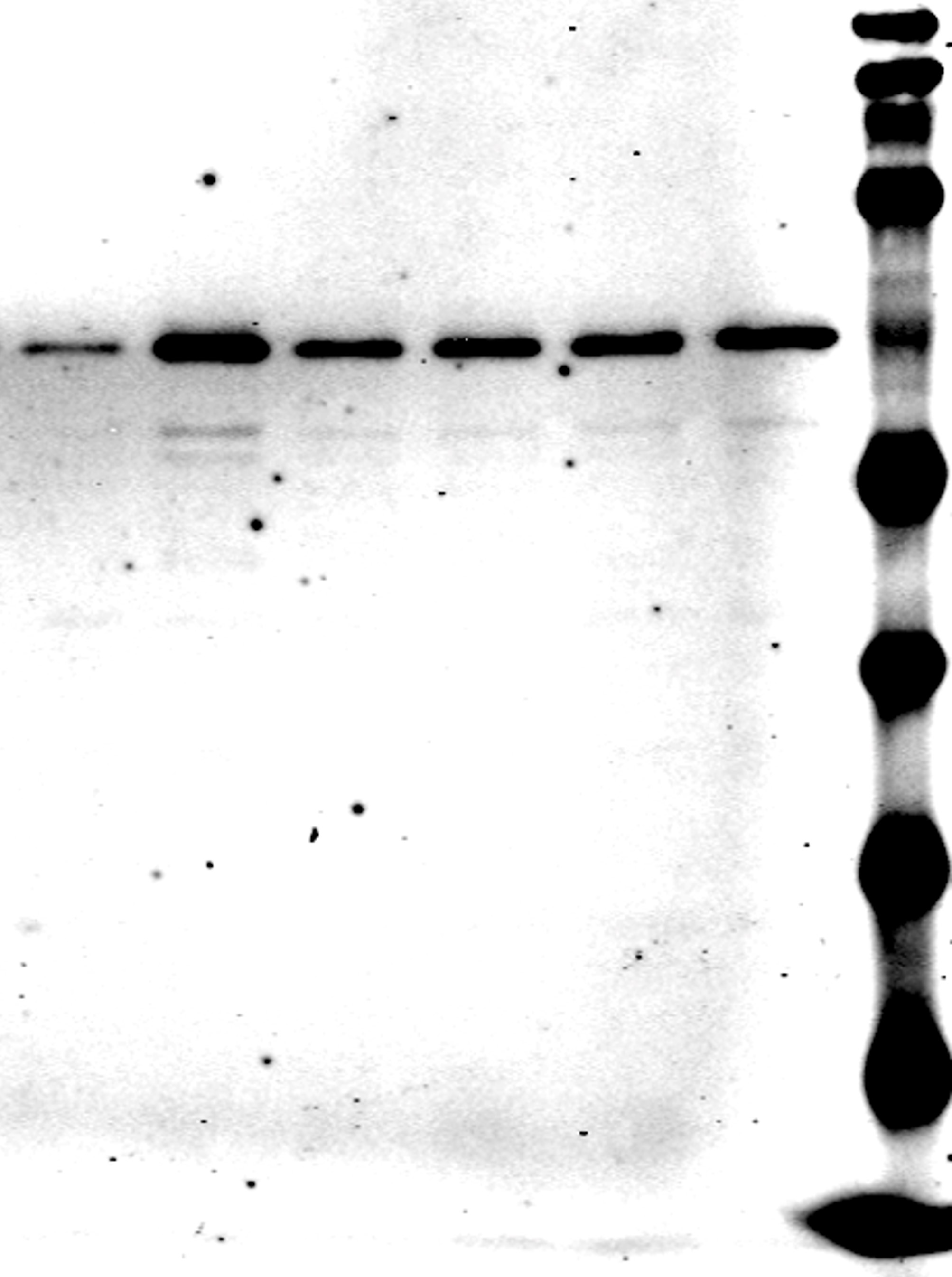

Supplement: Supplementary file 1 [file biomolecules-15-00034-s001.zip › Western blot raw data/Figure 6_pNF-kB_N2.jpg]

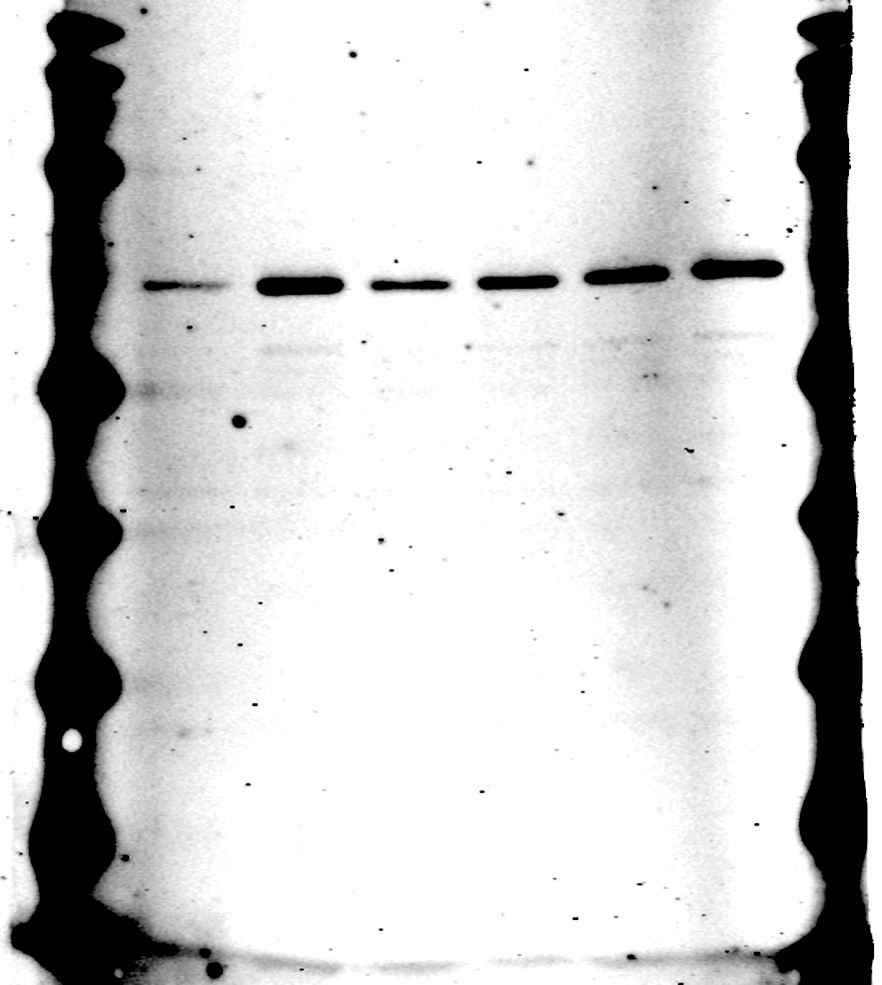

Supplement: Supplementary file 1 [file biomolecules-15-00034-s001.zip › Western blot raw data/Figure 6_pNF-kB_N3.jpg]

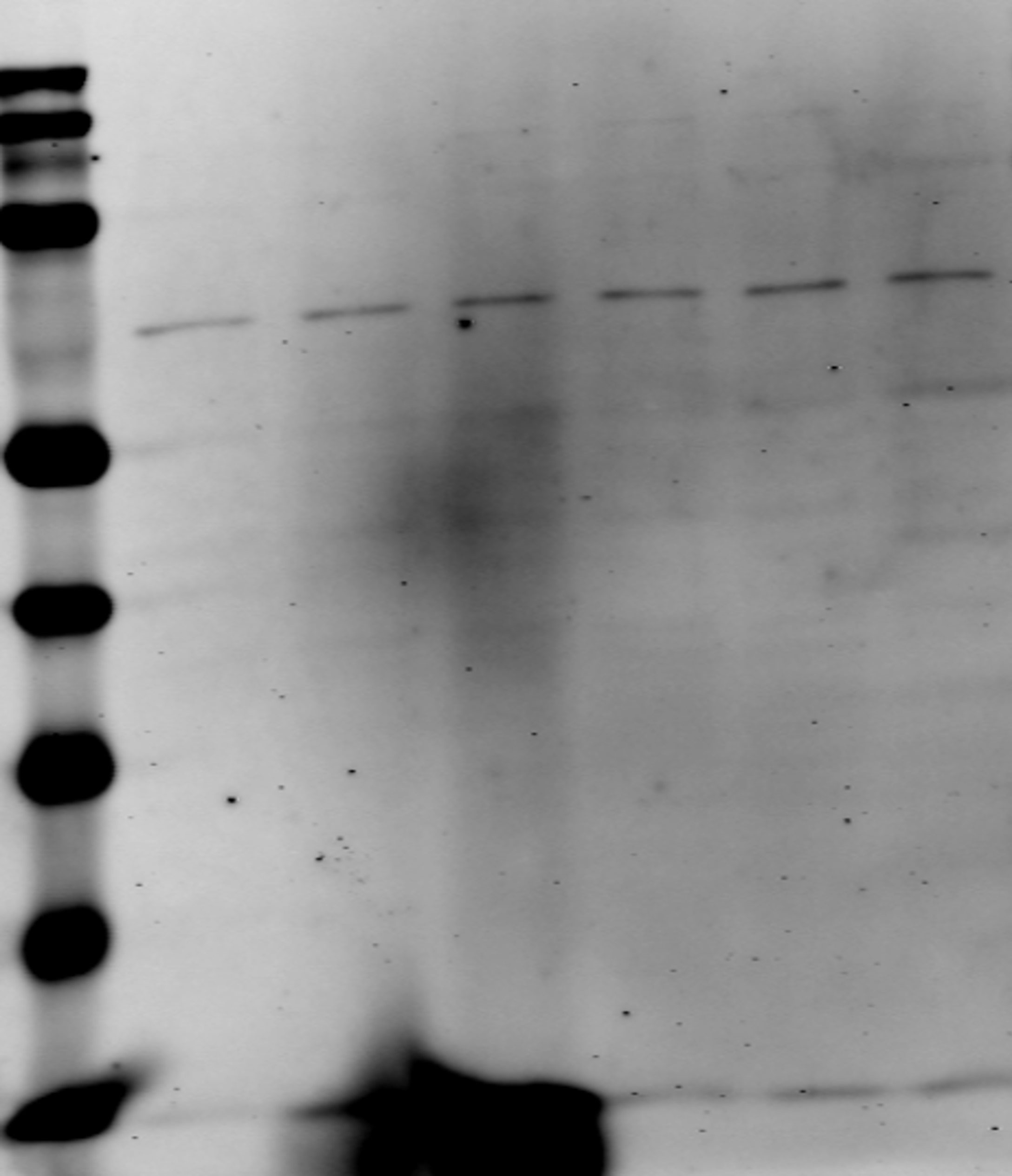

Supplement: Supplementary file 1 [file biomolecules-15-00034-s001.zip › Western blot raw data/Figure 6_Total NF-kB_N1.jpg]

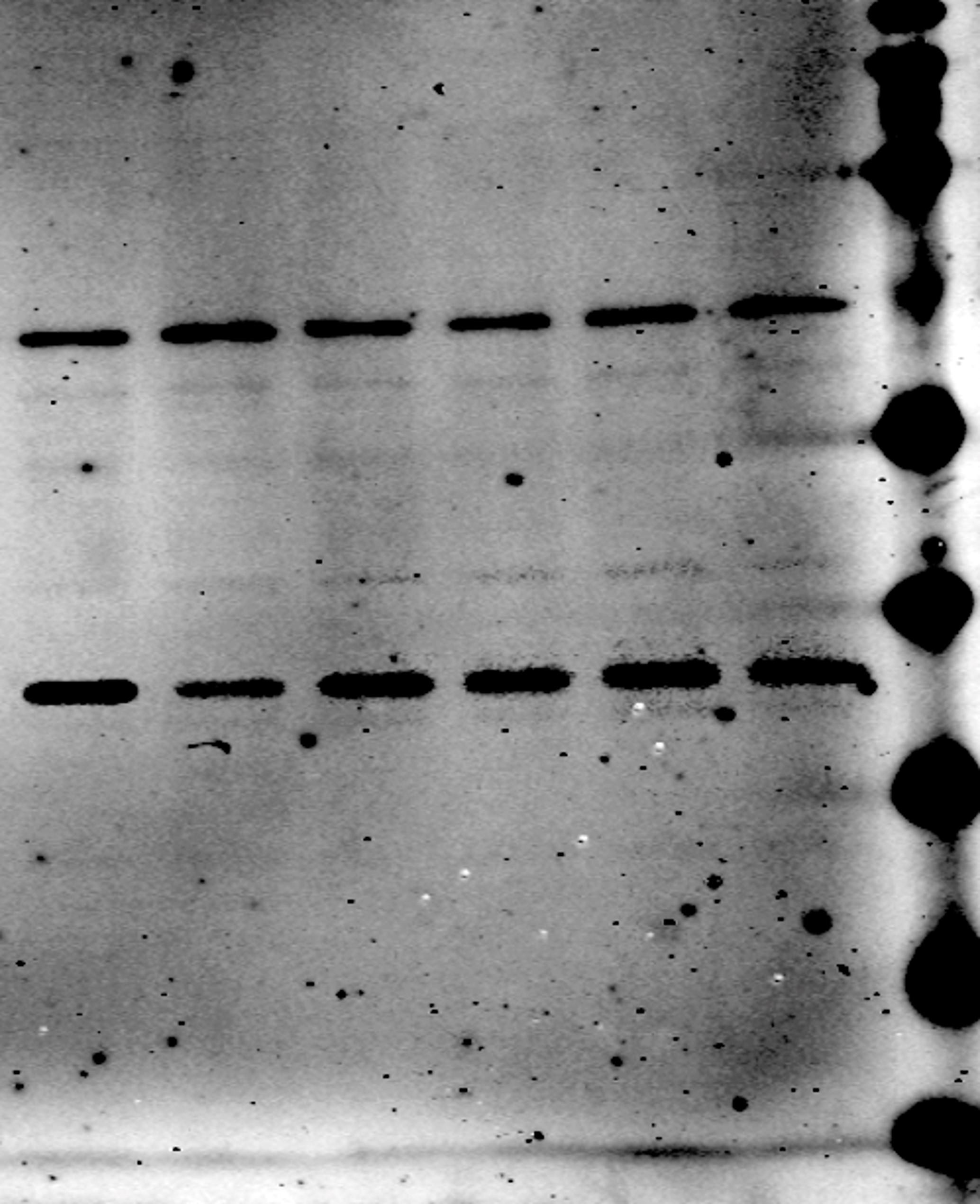

Supplement: Supplementary file 1 [file biomolecules-15-00034-s001.zip › Western blot raw data/Figure 6_Total NF-kB_N2.jpg]

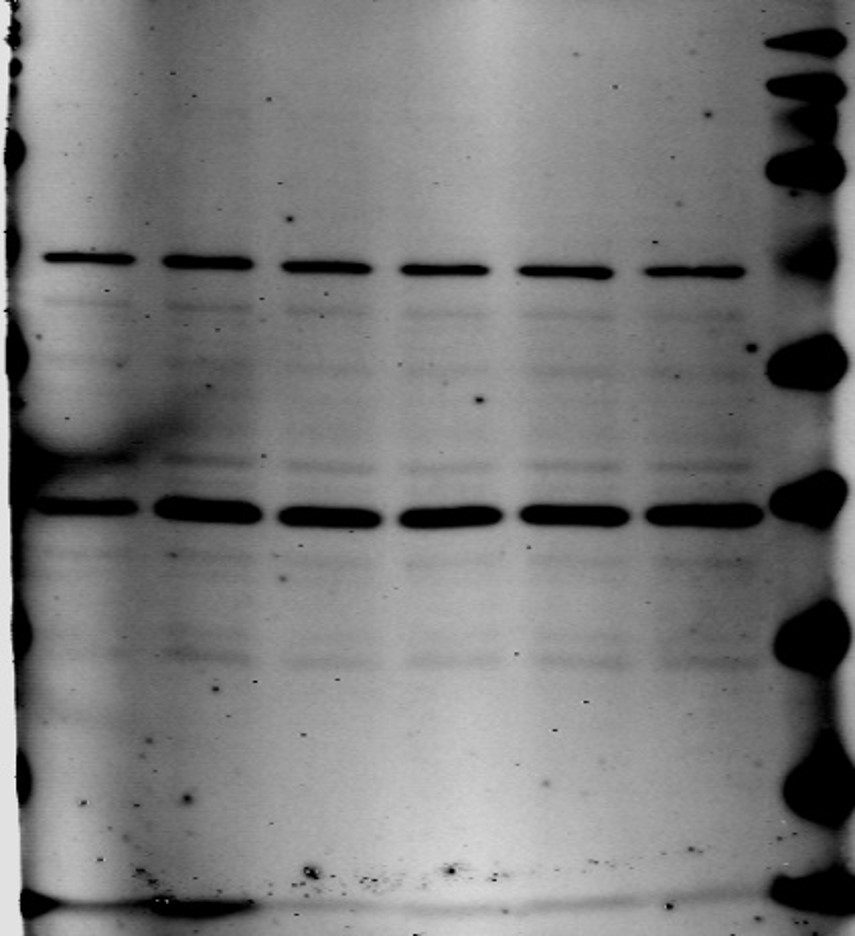

Supplement: Supplementary file 1 [file biomolecules-15-00034-s001.zip › Western blot raw data/Figure 6_Total NF-kB_N3.jpg]

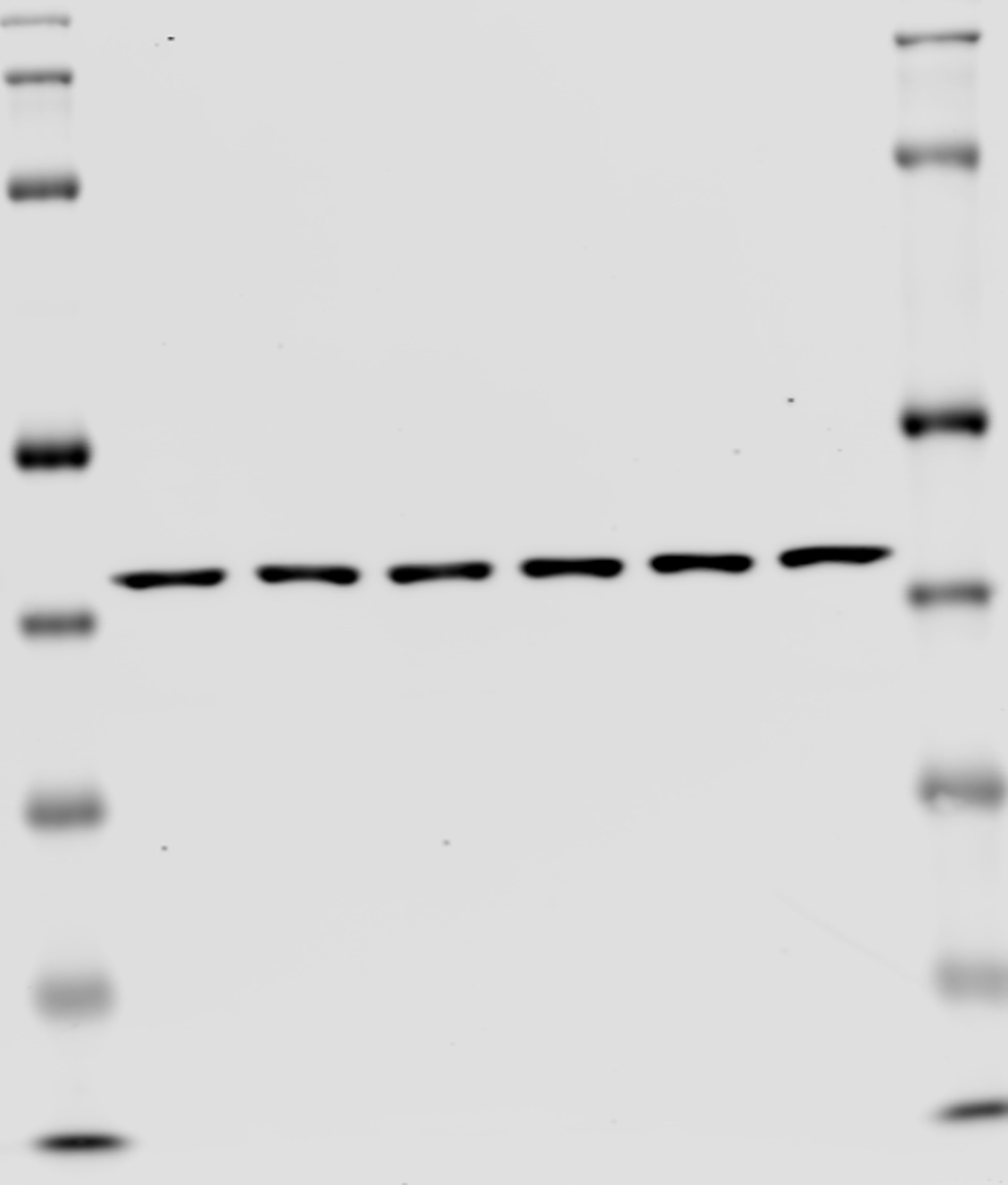

Supplement: Supplementary file 1 [file biomolecules-15-00034-s001.zip › Western blot raw data/Figure 7__Actin_N1.jpg]

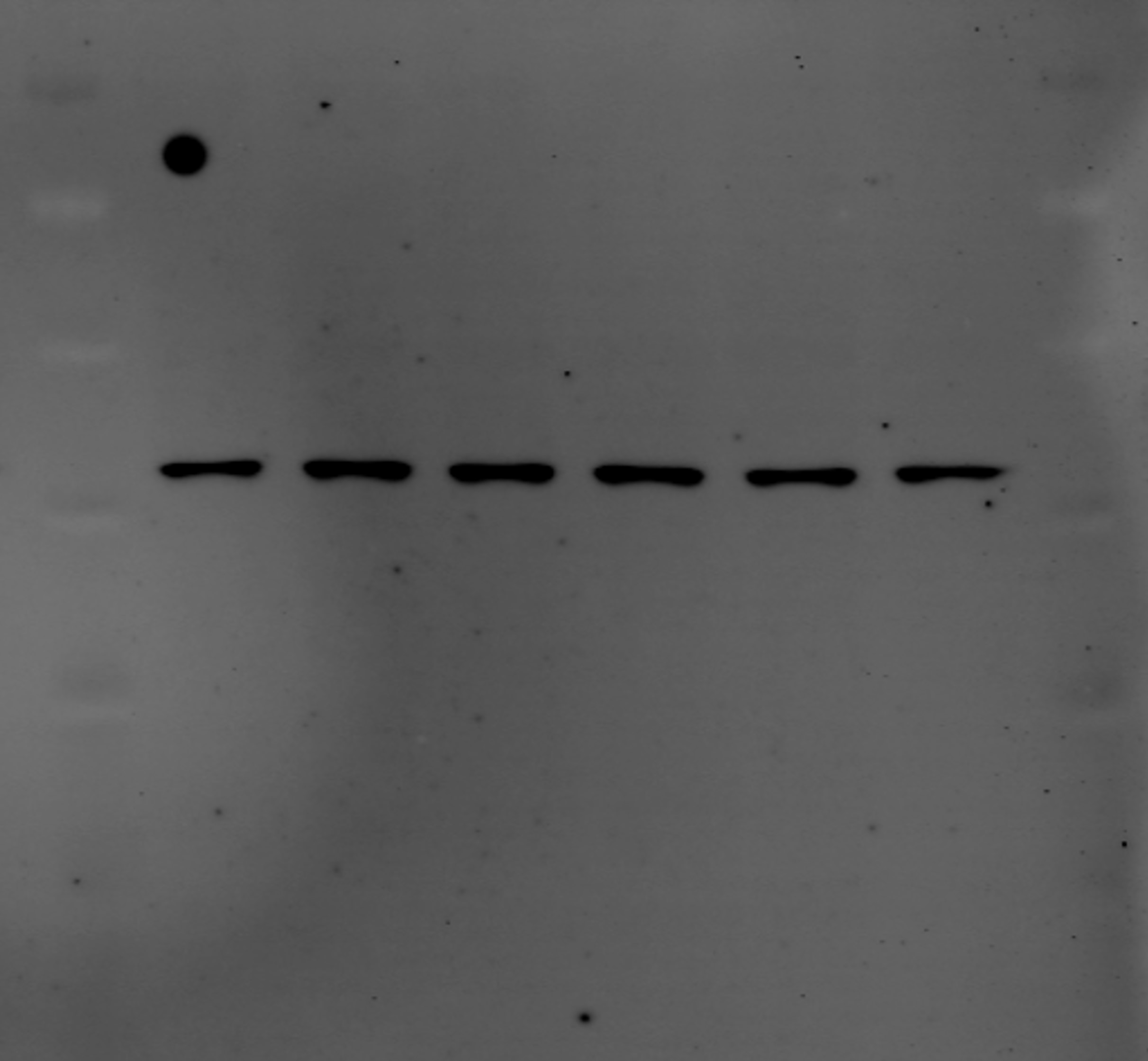

Supplement: Supplementary file 1 [file biomolecules-15-00034-s001.zip › Western blot raw data/Figure 7__Actin_N2.jpg]

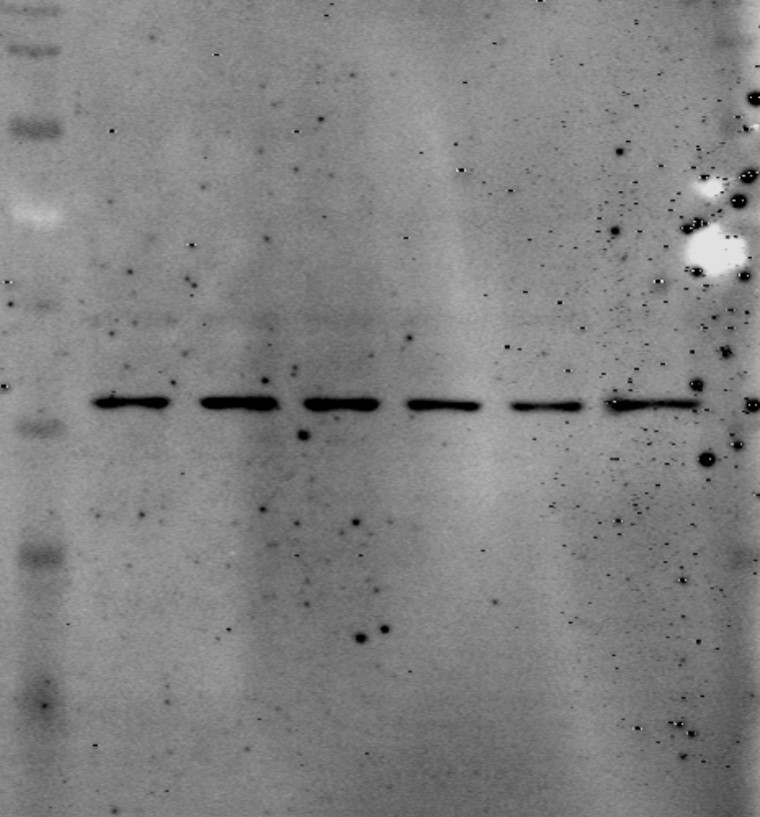

Supplement: Supplementary file 1 [file biomolecules-15-00034-s001.zip › Western blot raw data/Figure 7__Actin_N3.jpg]

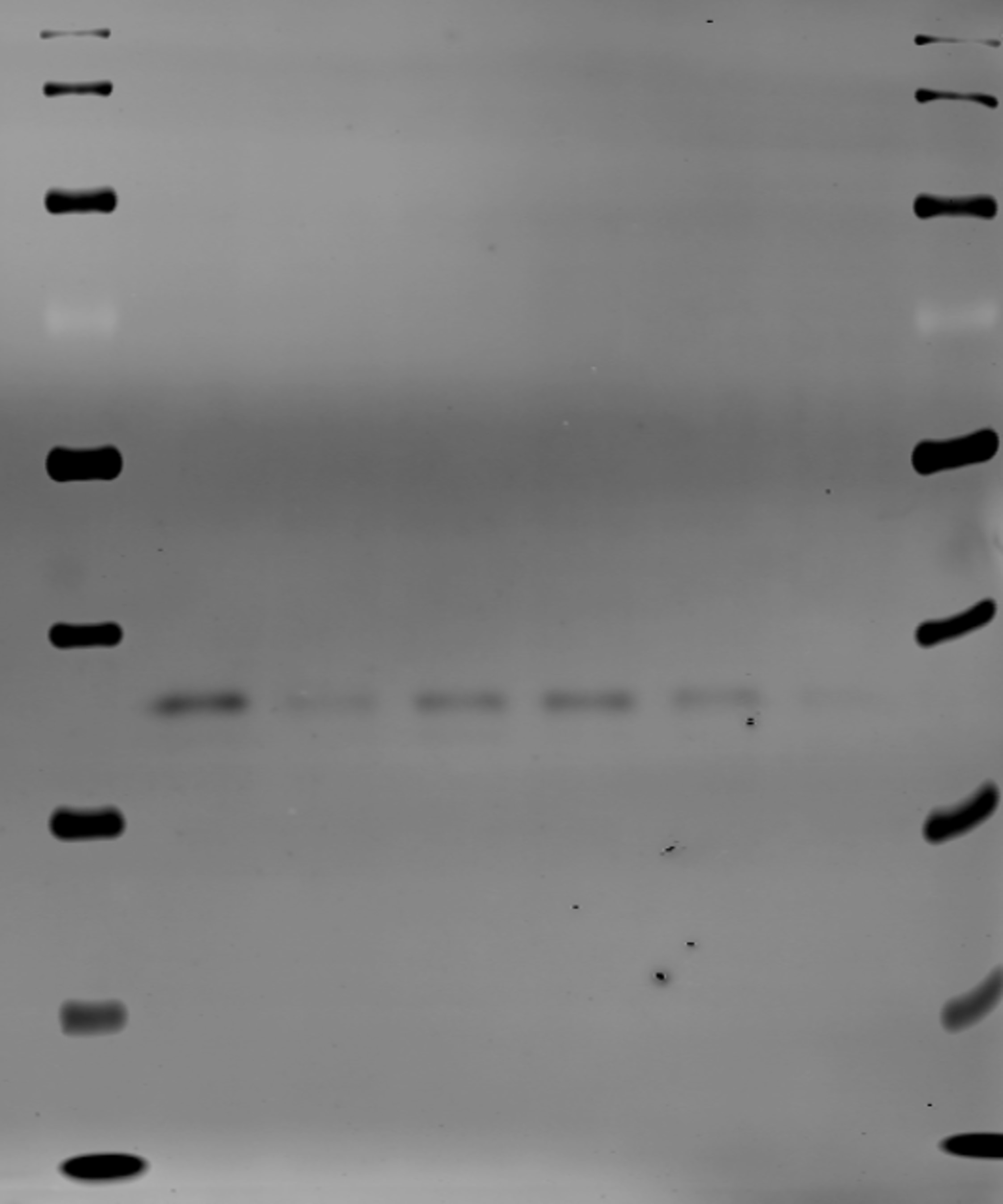

Supplement: Supplementary file 1 [file biomolecules-15-00034-s001.zip › Western blot raw data/Figure 7__IkB_N1.jpg]

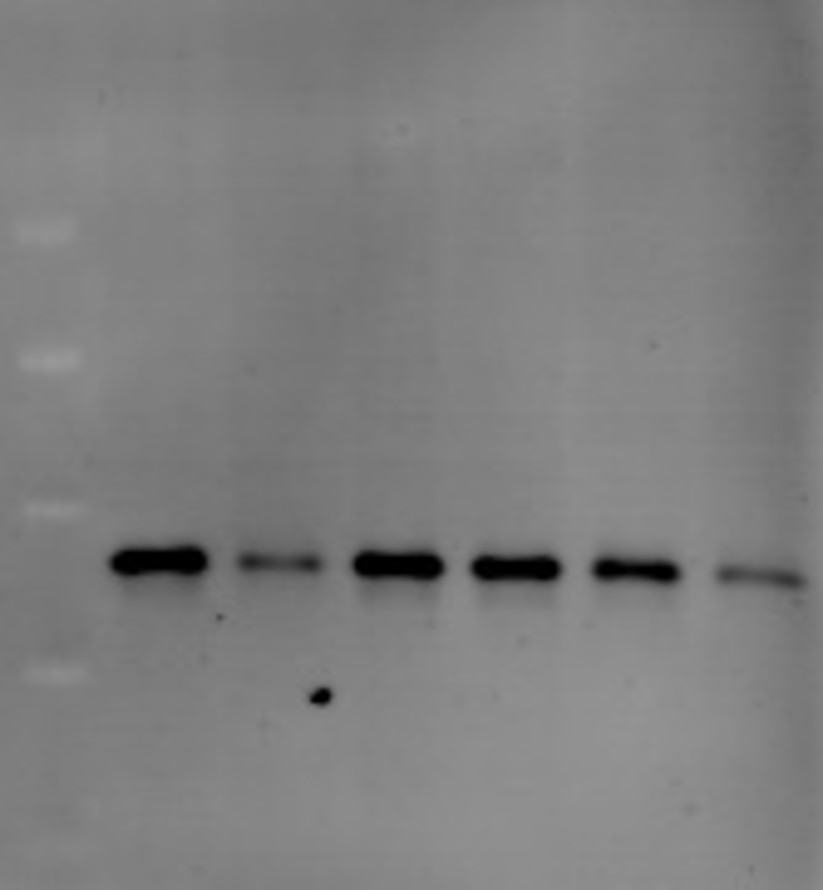

Supplement: Supplementary file 1 [file biomolecules-15-00034-s001.zip › Western blot raw data/Figure 7__IkB_N2.jpg]

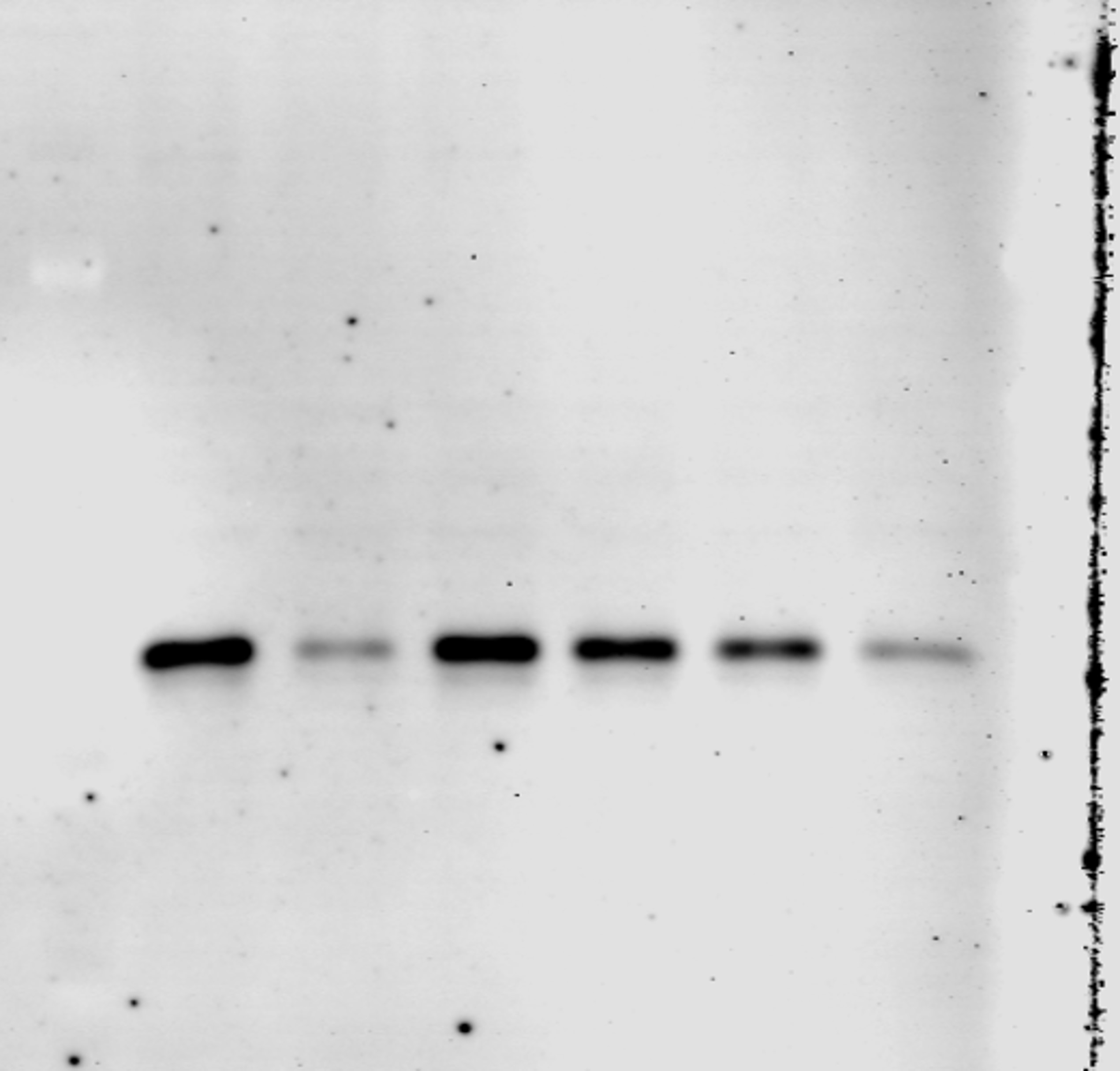

Supplement: Supplementary file 1 [file biomolecules-15-00034-s001.zip › Western blot raw data/Figure 7__IkB_N3.jpg]

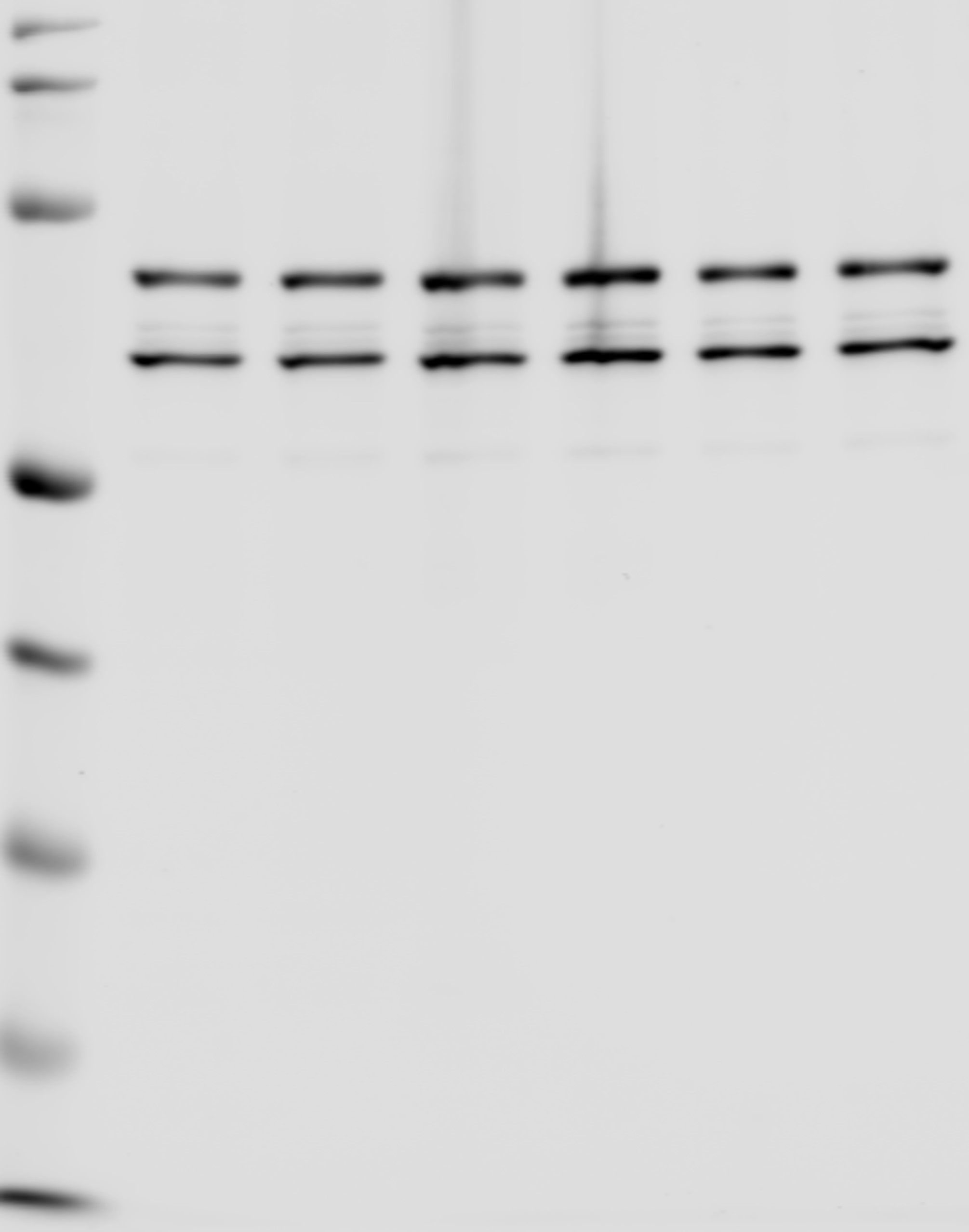

Supplement: Supplementary file 1 [file biomolecules-15-00034-s001.zip › Western blot raw data/Figure 7__Lamin A-C_N1.jpg]

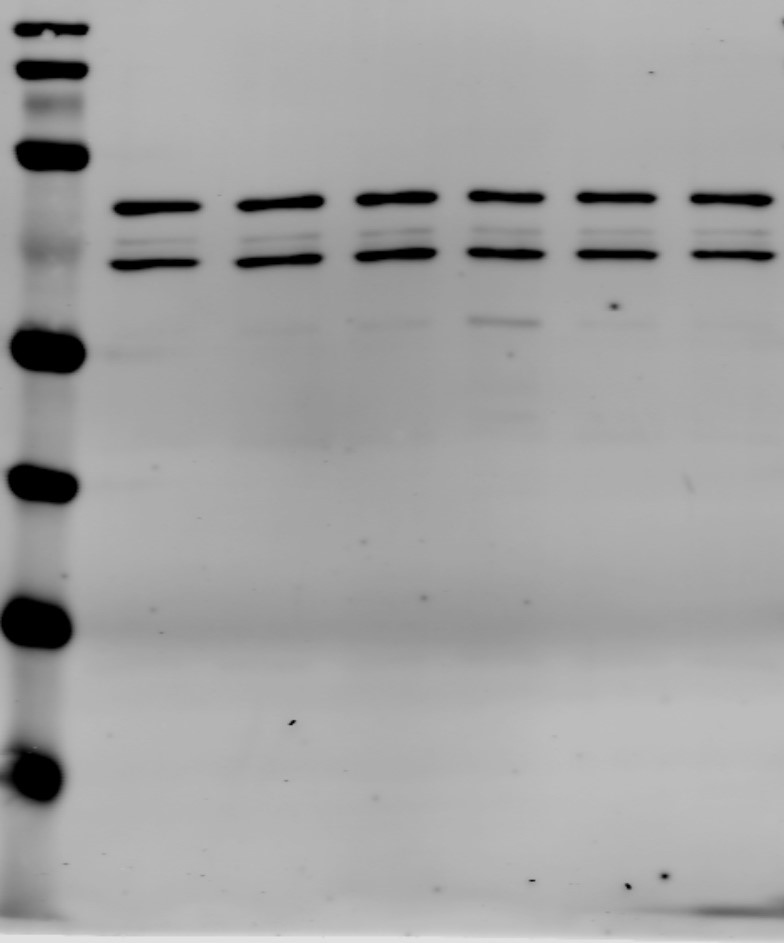

Supplement: Supplementary file 1 [file biomolecules-15-00034-s001.zip › Western blot raw data/Figure 7__Lamin A-C_N2.jpg]

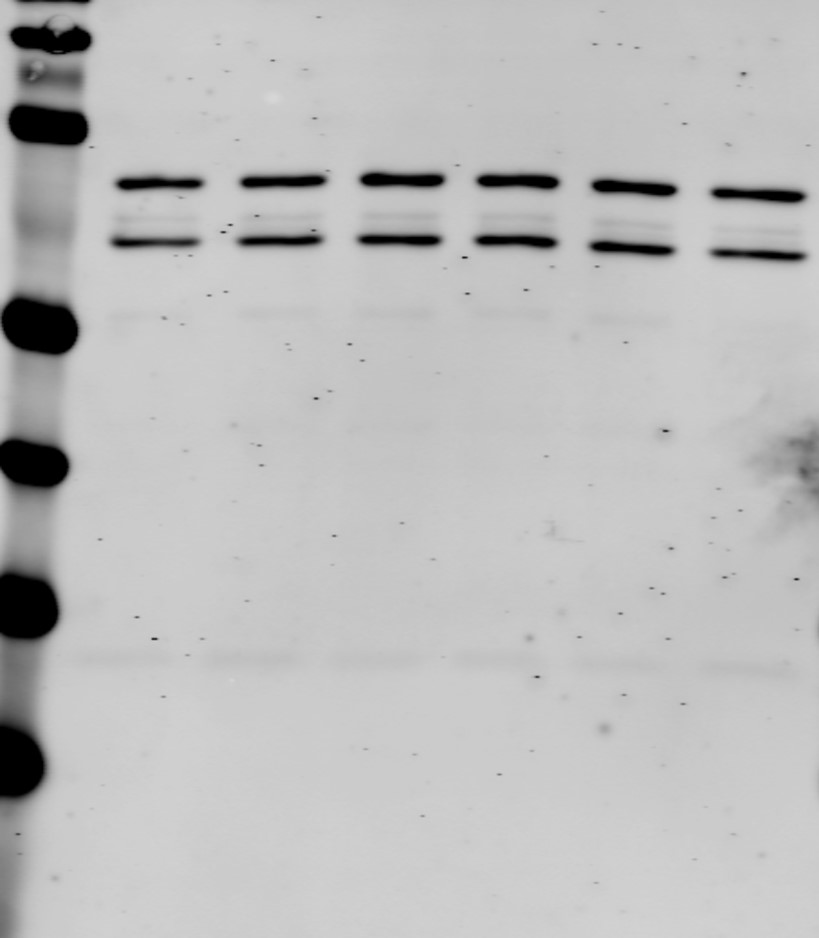

Supplement: Supplementary file 1 [file biomolecules-15-00034-s001.zip › Western blot raw data/Figure 7__Lamin A-C_N3.jpg]

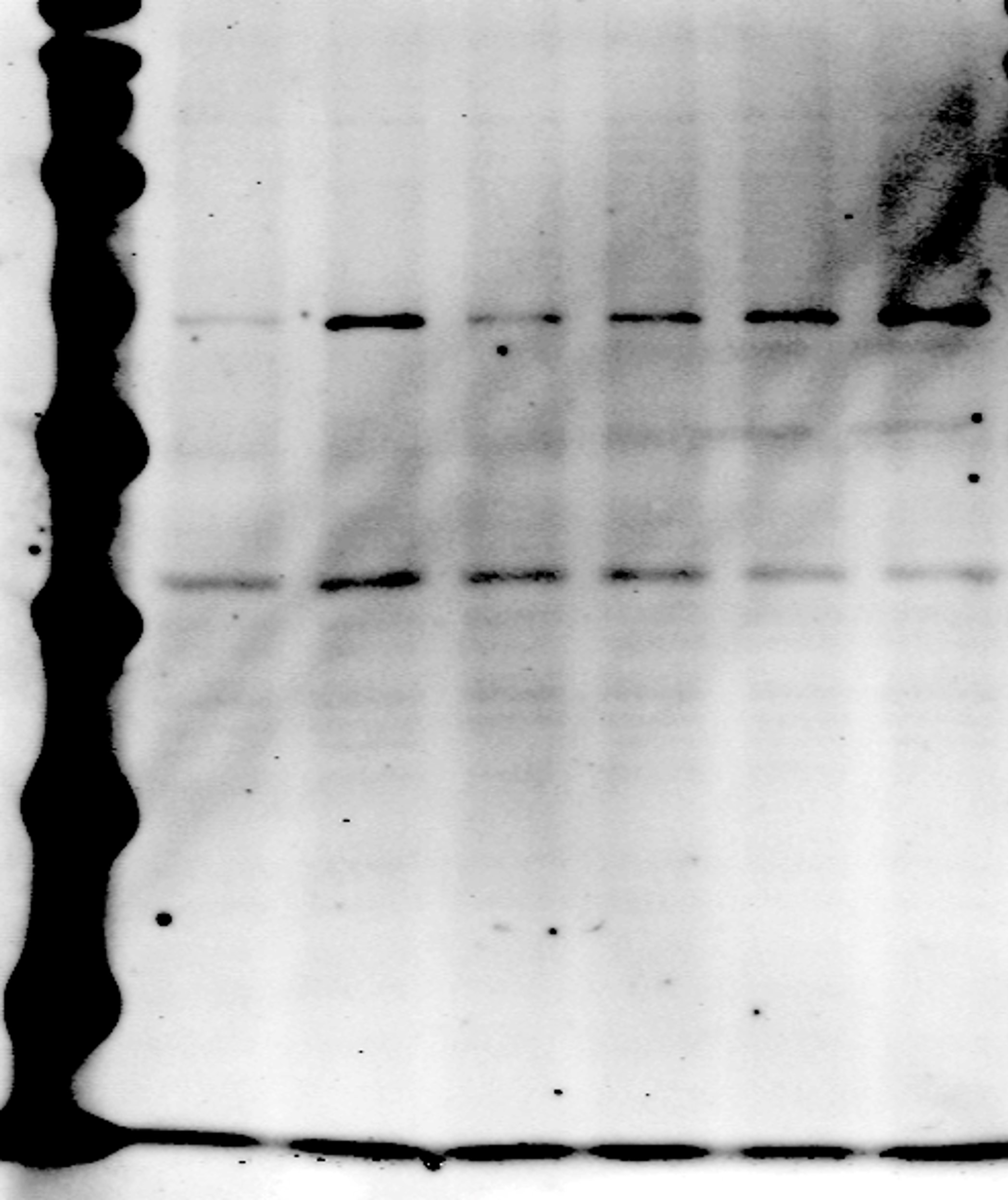

Supplement: Supplementary file 1 [file biomolecules-15-00034-s001.zip › Western blot raw data/Figure 7_NFkB_N1.jpg]

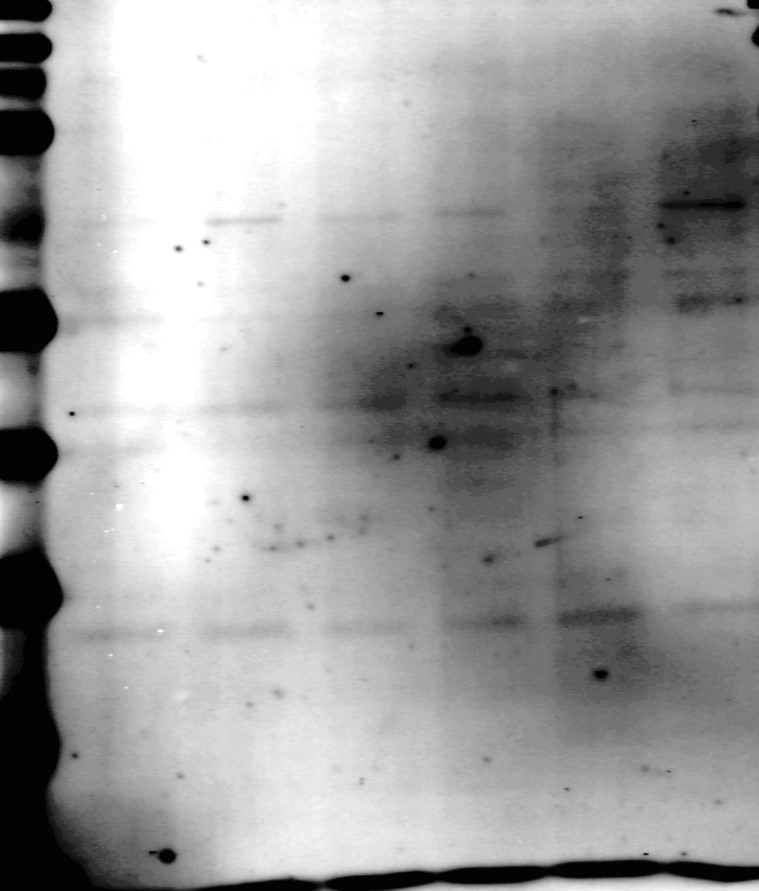

Supplement: Supplementary file 1 [file biomolecules-15-00034-s001.zip › Western blot raw data/Figure 7_NFkB_N2.jpg]

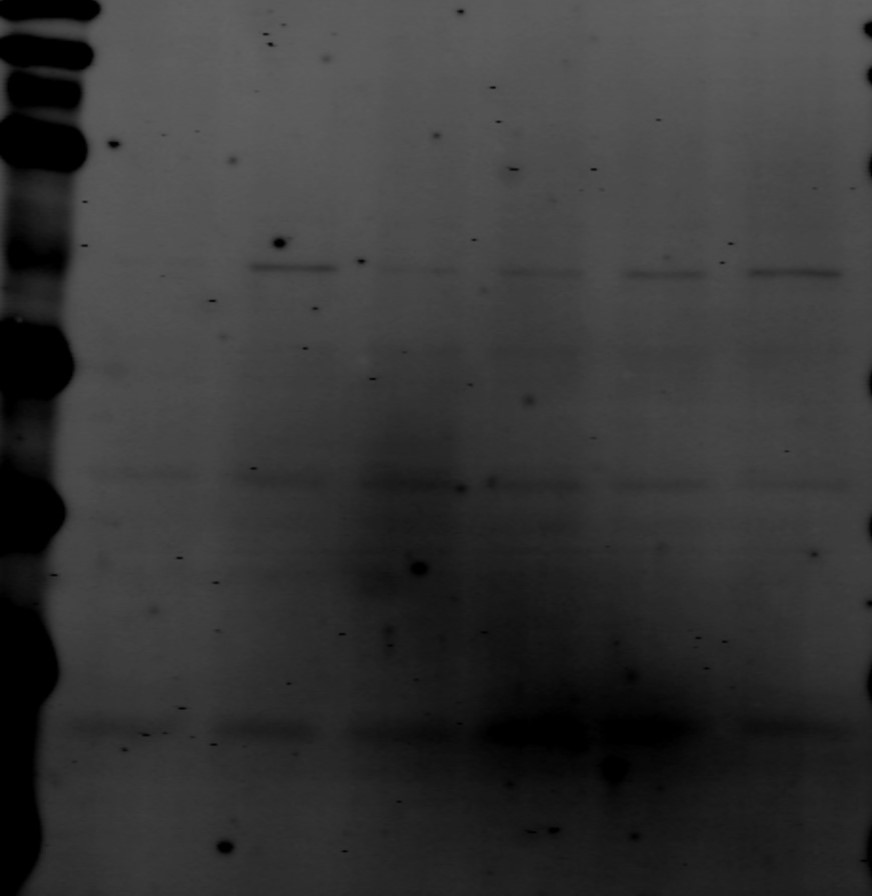

Supplement: Supplementary file 1 [file biomolecules-15-00034-s001.zip › Western blot raw data/Figure 7_NFkB_N3.jpg]

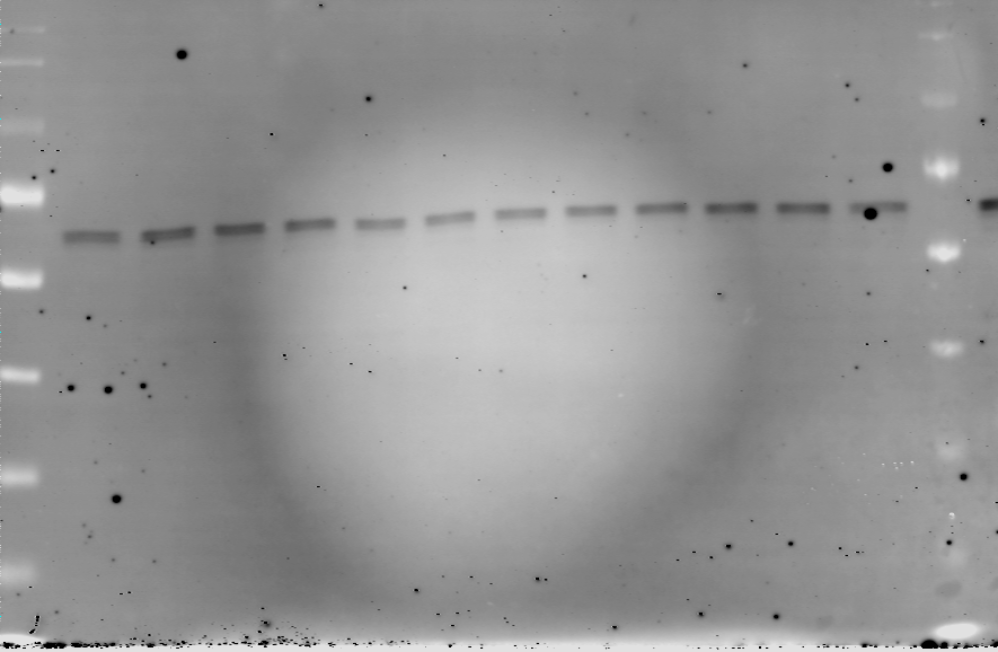

Supplement: Supplementary file 1 [file biomolecules-15-00034-s001.zip › Western blot raw data/Figure 8_ Total AKT_N1-N2.jpg.png]

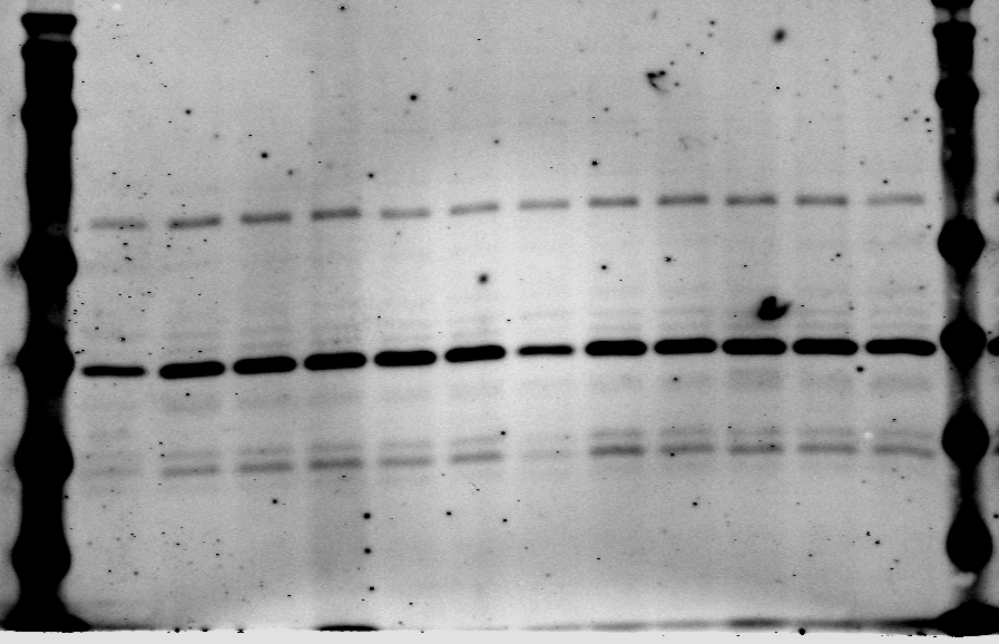

Supplement: Supplementary file 1 [file biomolecules-15-00034-s001.zip › Western blot raw data/Figure 8_pAKT_N1-N2.jpg]

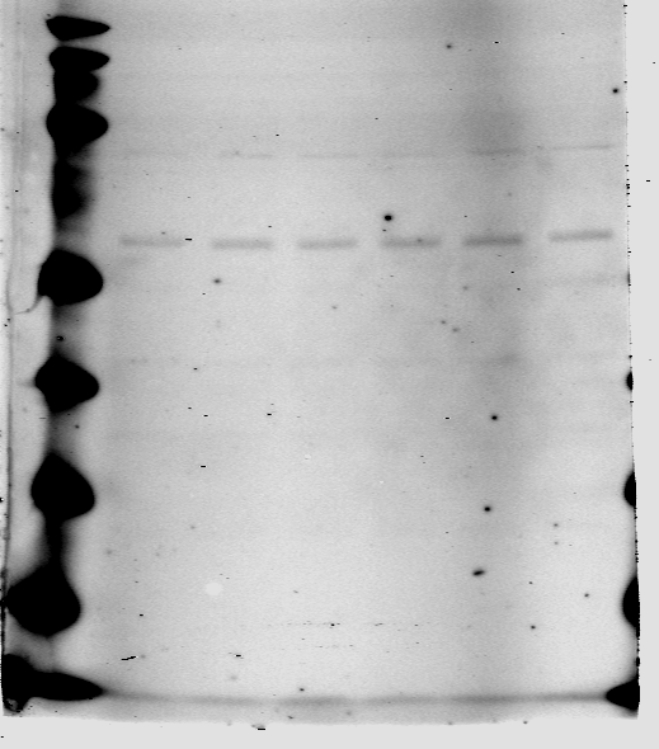

Supplement: Supplementary file 1 [file biomolecules-15-00034-s001.zip › Western blot raw data/Figure 8_pAKT_N3.jpg]

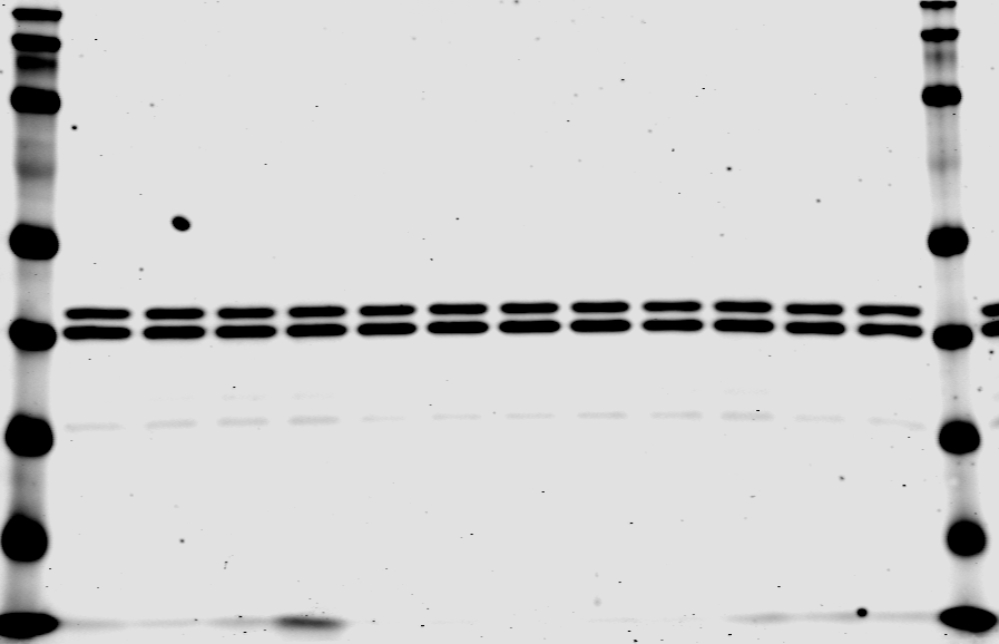

Supplement: Supplementary file 1 [file biomolecules-15-00034-s001.zip › Western blot raw data/Figure 8_pERK1-2_N1-N2.jpg]

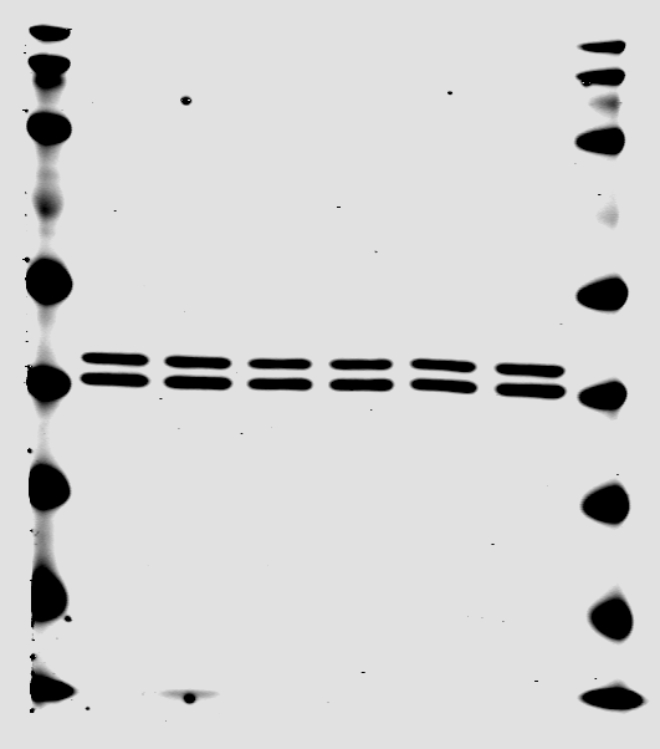

Supplement: Supplementary file 1 [file biomolecules-15-00034-s001.zip › Western blot raw data/Figure 8_pERK1-2_N3.jpg]

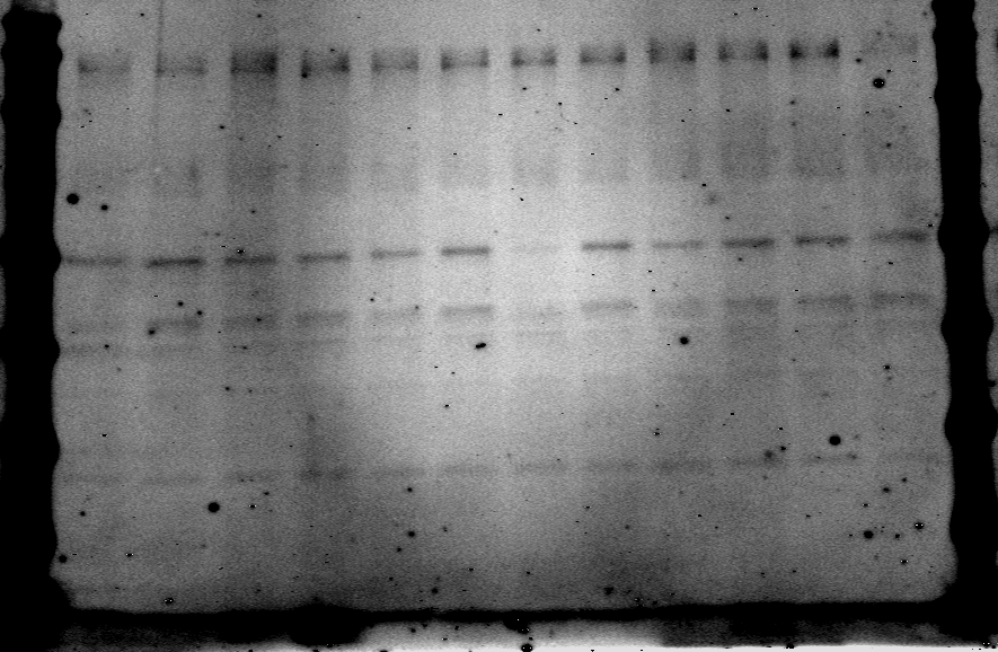

Supplement: Supplementary file 1 [file biomolecules-15-00034-s001.zip › Western blot raw data/Figure 8_pJNK_N1-N2.jpg]

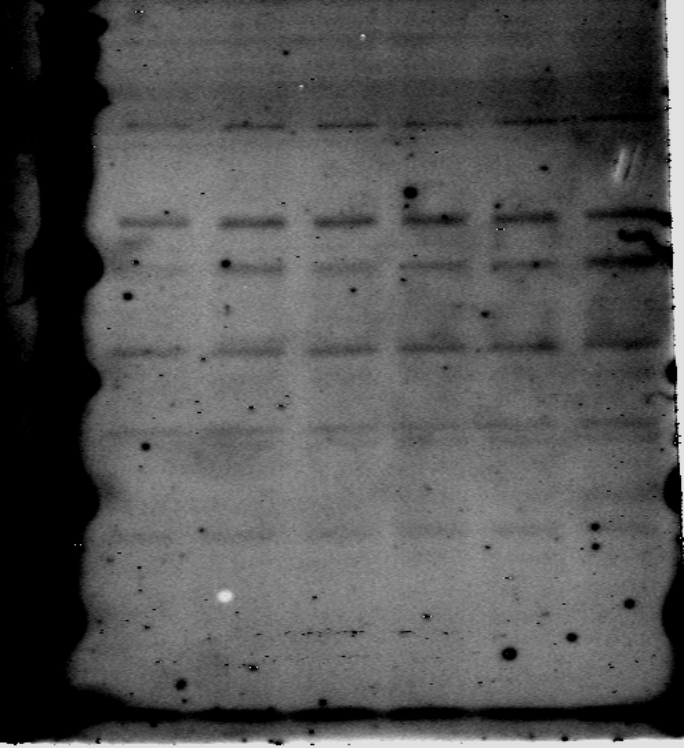

Supplement: Supplementary file 1 [file biomolecules-15-00034-s001.zip › Western blot raw data/Figure 8_pJNK_N3.jpg]

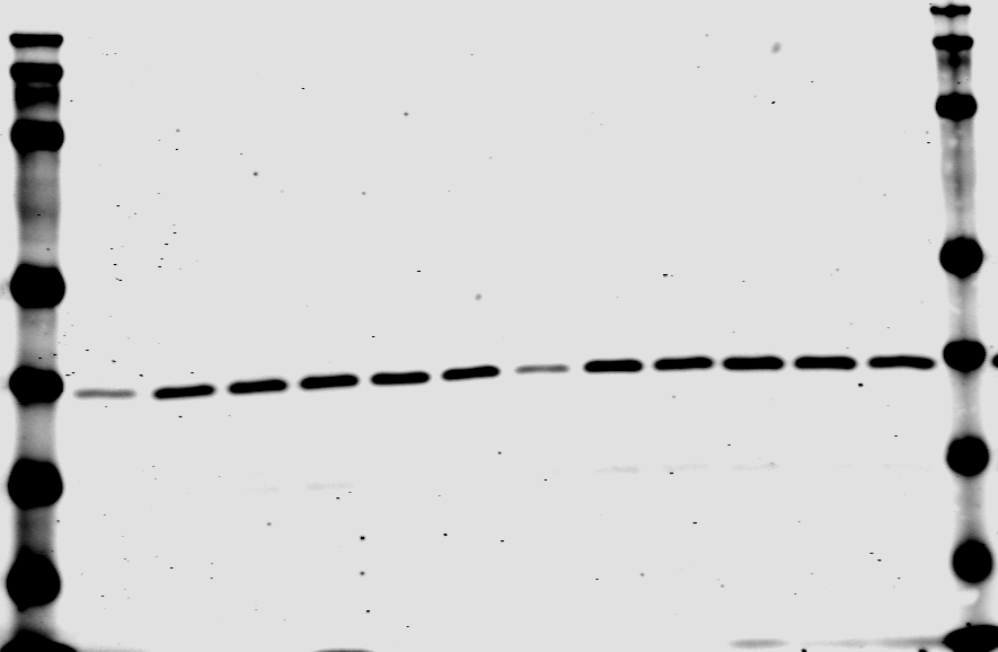

Supplement: Supplementary file 1 [file biomolecules-15-00034-s001.zip › Western blot raw data/Figure 8_pp38_N1-N2.jpg]

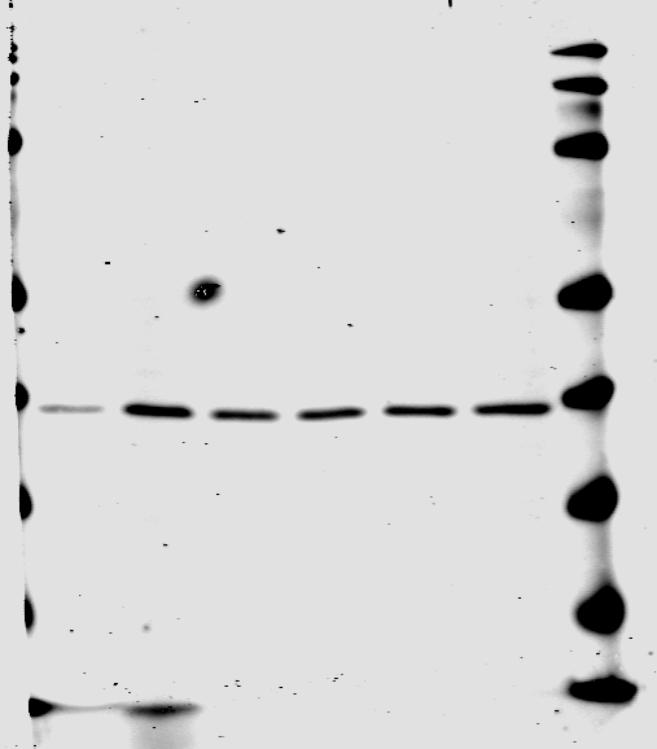

Supplement: Supplementary file 1 [file biomolecules-15-00034-s001.zip › Western blot raw data/Figure 8_pp38_N3.jpg.jpg]

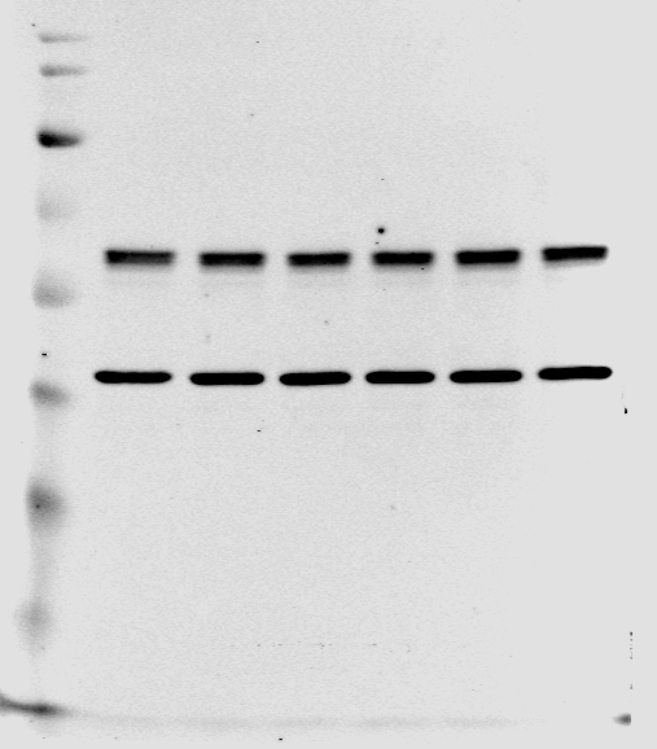

Supplement: Supplementary file 1 [file biomolecules-15-00034-s001.zip › Western blot raw data/Figure 8_Total AKT_N3.jpg.jpg]

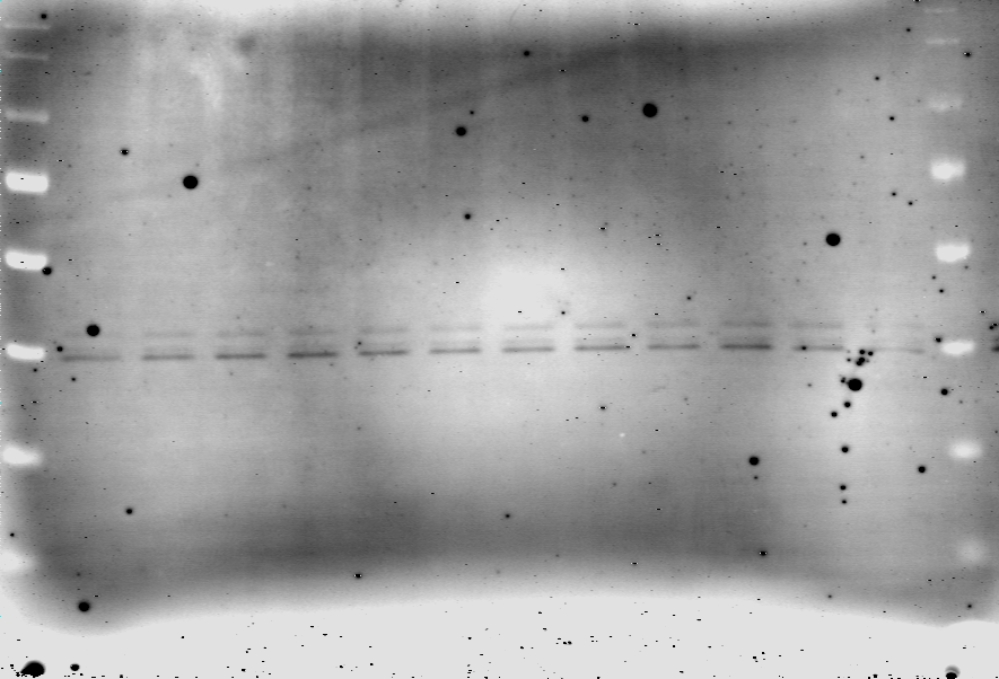

Supplement: Supplementary file 1 [file biomolecules-15-00034-s001.zip › Western blot raw data/Figure 8_Total ERK_N1-N2.jpg.jpg]

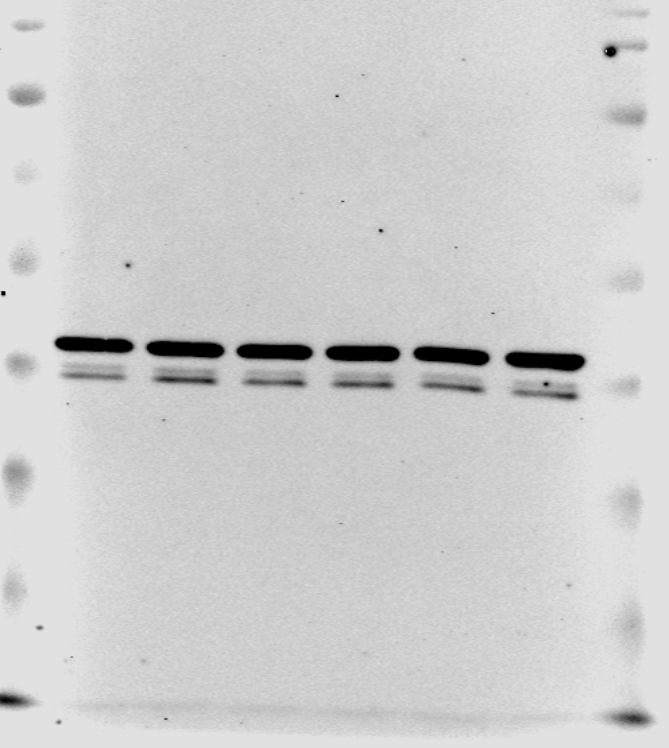

Supplement: Supplementary file 1 [file biomolecules-15-00034-s001.zip › Western blot raw data/Figure 8_Total ERK1-2_N3.jpg.jpg]

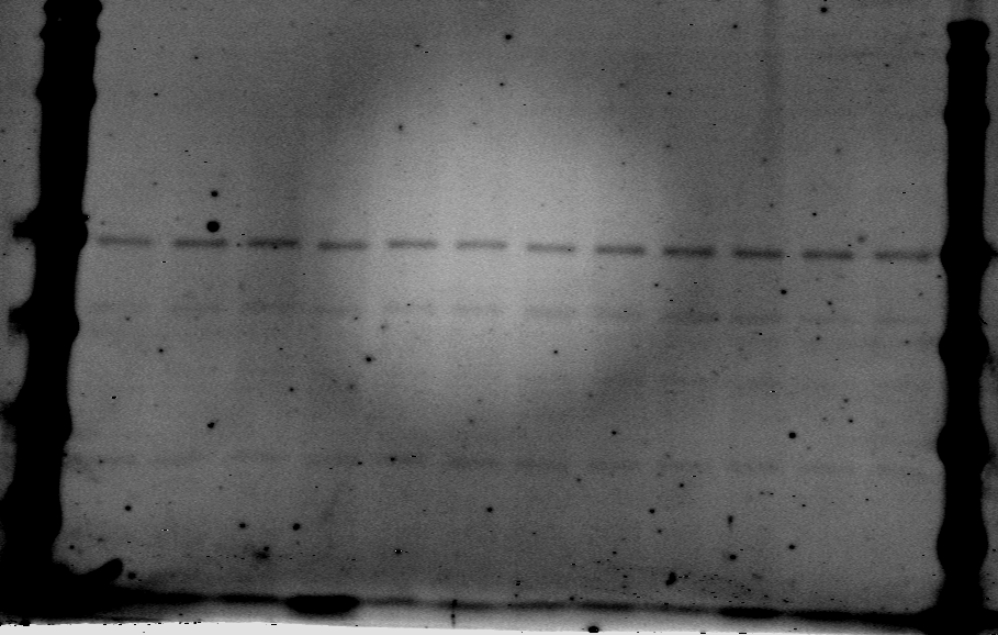

Supplement: Supplementary file 1 [file biomolecules-15-00034-s001.zip › Western blot raw data/Figure 8_Total JNK_N1-N2.jpg]

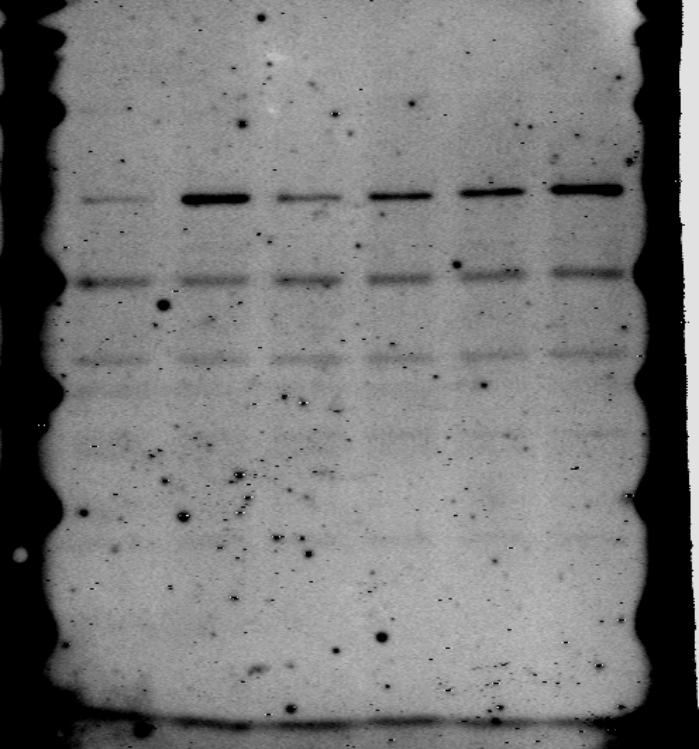

Supplement: Supplementary file 1 [file biomolecules-15-00034-s001.zip › Western blot raw data/Figure 8_Total JNK_N3.jpg]

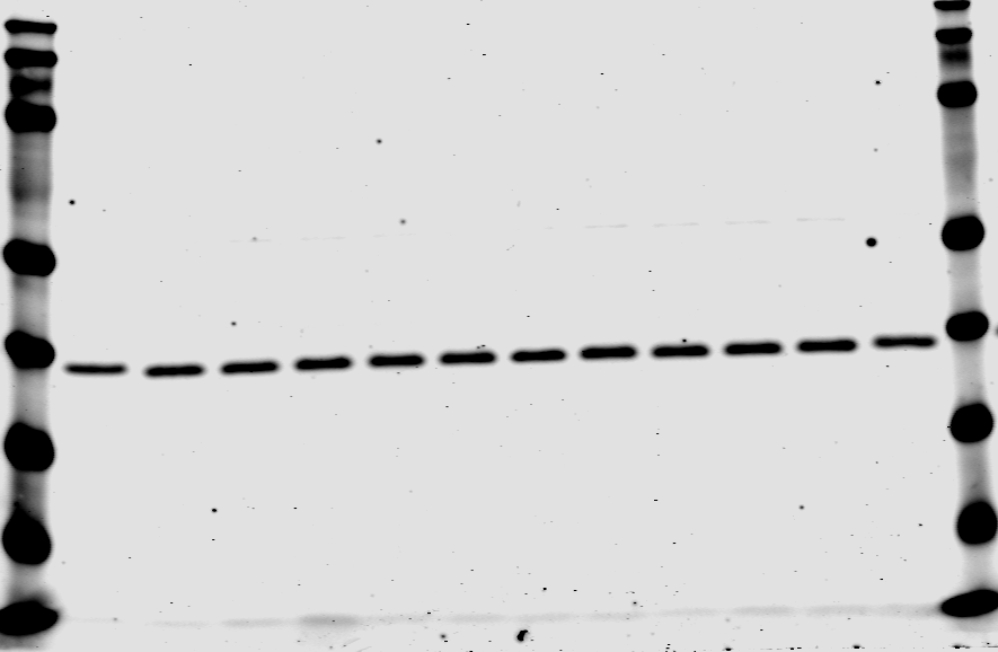

Supplement: Supplementary file 1 [file biomolecules-15-00034-s001.zip › Western blot raw data/Figure 8_Total p38_N1-N2.jpg]

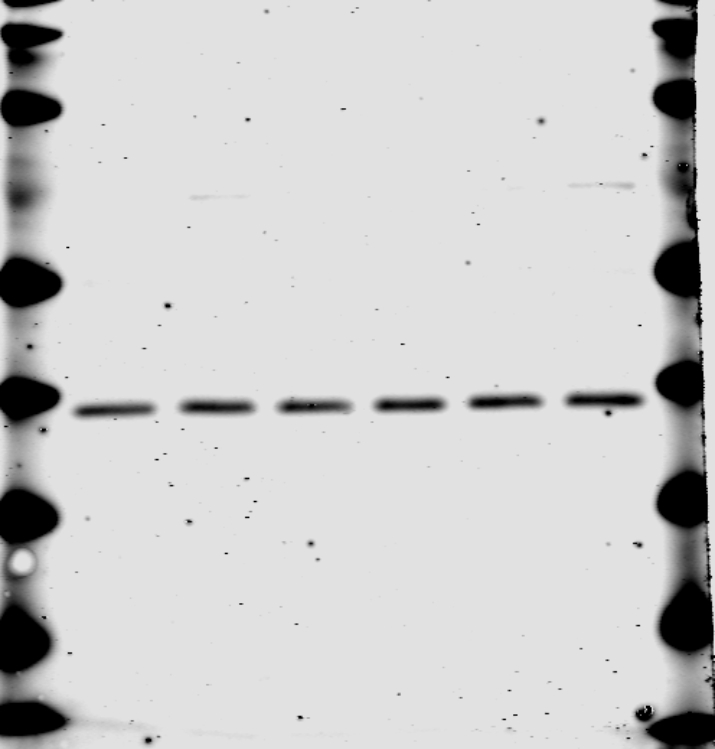

Supplement: Supplementary file 1 [file biomolecules-15-00034-s001.zip › Western blot raw data/Figure 8_Total p38_N3.jpg]
